# Behavioral and brain-wide neural signatures of sundowning in Alzheimer’s disease

**DOI:** 10.21203/rs.3.rs-9272255/v1

**Published:** 2026-04-20

**Authors:** Michelle Jin, Yujing Fu, Xinyue Chen, Holly C. Hunsberger, Sophia Sorid, Gabriella Mouris, Connie Huang, Alicia R. Whye, Simon O. Ogundare, Yifan Yao, Shannon C. Shipley, Ina Pavlova, Rae Silver, Seonjoo Lee, Yueqing Peng, Christine. A. Denny

**Affiliations:** 1 Medical Scientist Training Program (MSTP), Columbia University Irving Medical Center (CUIMC), New York, NY, 10032, USA; 2 Neurobiology and Behavior (NB&B) Graduate Program, Columbia University, New York, NY, 10027, USA; 3 Department of Biostatistics, Mailman School of Public Health, Columbia University, New York, NY, USA; 4 Department of Psychiatry, Columbia University Irving Medical Center (CUIMC), New York, NY, 10032, USA; 5 Division of Systems Neuroscience, New York State Psychiatric Institute (NYSPI) dba Research Foundation for Mental Hygiene, Inc. (RFMH), New York, NY, 10032, USA; 6 Current Institution: Center for Neurodegenerative Disease and Therapeutics, Rosalind Franklin University of Medicine and Science, Chicago, IL; 7 Columbia College, New York, NY, 10027, USA; 8 Barnard College, New York, NY, 10027, USA; 9 Current Institution: Tri-Institutional MD-PhD Program, Weill Cornell Medicine, New York, NY, 10065, USA; 10 Current Institution: Children’s Hospital of Philadelphia, Philadelphia, PA 19104, USA; 11 Current Institution: Hamamatsu Photonics, Bridgewater, NJ, 08807, USA; 12 Department of Psychology, Columbia University, New York, NY, 10027, USA; 13 Department of Neuroscience & Behavior, Barnard College, New York, NY, 10027, USA; 14 Area Brain Aging and Mental Health, New York State Psychiatric Institute, New York, NY, USA; 15 Division of Mental Health Data Science, New York State Psychiatric Institute, New York, NY, 10032 USA.; 16 Department of Neurology, Vagelos College of Physicians and Surgeons, Columbia University, New York, 10032, USA; 17 Department of Pathology and Cell Biology, Vagelos College of Physicians and Surgeons, Columbia University, New York, 10032, USA

**Keywords:** immediate early gene, Arc/Arg3.1, APP/PS1, sleep-wake, anxiety, nighttime wandering

## Abstract

Sundowning, a common yet poorly understood neuropsychiatric syndrome in Alzheimer’s disease (AD), manifests as evening-specific increases in agitation, confusion, and anxiety. Despite its prevalence and contribution to patient distress, its neural mechanisms remain elusive. Here, we establish a preclinical model of sundowning by characterizing sleep-wake, behavioral, and network-level alterations in an AD mouse model. Aged AD mice exhibit disrupted sleep-wake patterns and reduced slow-wave sleep. Behavioral and pose-tracking analyses revealed motor agitation and a distinct sundowning-like behavioral fingerprint selectively at Sundown. The suprachiasmatic nucleus (SCN) showed disruptions in time-of-day-dependent activation of vasopressin-expressing cells and brain-wide activity-dependent tagging identified hyperconnectivity amongst sensorimotor regions in AD mice at Sundown. Resting-state fMRI data from the Alzheimer’s Disease Neuroimaging Initiative revealed analogous Salience Network alterations in AD subjects. Overall, these cross-species findings define a systems-level framework for sundowning and highlight regions that may be targeted to alleviate a debilitating symptom of AD.

## Introduction

Alzheimer’s disease (AD), the leading cause of dementia worldwide^1^, poses substantial socioeconomic and emotional burdens on patients, caregivers, and the healthcare system. Although progressive memory loss defines AD clinically, up to two-thirds of patients experience profound disturbances in sleep and circadian rhythms^2^. Mounting evidence supports a bidirectional relationship between sleep disruption and AD pathophysiology^3-9^. For example, in humans, decreased non-rapid eye movement (NREM) sleep is associated with impaired cognitive performance and higher levels of amyloid beta (Aβ) and tau pathology in the cortex during prodromal stages of AD^10,11^. In mice, soluble Aβ levels in the brain fluctuate with the sleep—wake cycle^4^, and the accumulation of Aβ in transgenic mice disrupts normal sleep-wake rhythms^8^. Additionally, sleep deprivation increases Aβ and tau pathology, whereas sleep stimulation, particularly of slow-wave activity (SWA), decreases pathology^4,12^.

Despite this literature, relatively little is known about sundown syndrome (“sundowning”), the increased prevalence or severity of neuropsychiatric symptoms (NPS) during the evening or transition to evening^13-17^. Sundowning prevalence varies widely, ranging up to 66% in dementia patients^18^ and symptoms encompass agitation, confusion, wandering, aggression, anxiety, and delusions^13^. Moreover, sundowning is associated with dementia severity^15,19^ and the apolipoprotein E (APOE) ε4 allele, the predominate genetic risk factor for late-onset AD^20^. While involvement of circadian and brainstem regions governing sleep-wake and arousal-attention processes has been implicated^21,22^, the neural mechanisms of sundowning remain poorly understood.

Here, we investigate sundowning-like behavior and the underlying neural correlates using multiple levels of investigation in the APPswe/PSEN1δE9 (AD) mouse model^23^. We report steeper degradation of sleep-wake rhythms with aging, alongside NREM disruptions which correlate with pathology in AD mice. Applying standard and unsupervised machine learning strategies to analyze behavior at the beginning (Sunrise) and end (Sundown) of the active period, we identify a novel aberrant phenotype expressed prominently in aged AD mice at Sundown. Mapping the brain-wide activity patterns underlying this phenotype with the ArcCreER^T2^ mice and the SMARTTR package^24,25^, we identify a hyperconnectivity signature in AD mice amongst predominately isocortical sensorimotor regions. Finally, in human AD subjects, we find an analogous time-of-day-dependent functional connectivity signature in the Salience Network (SN) and highlight the anterior cingulate area (ACA) and prelimbic areas (PL) as overlapping nodes from our mouse brain-mapping results and as possible therapeutic targets.

## Results

### Steeper sleep-wake rhythm degradation and disrupted slow-wave activity correlate with pathology burden in AD mice

**Extended Data Fig. 1 ∣:**
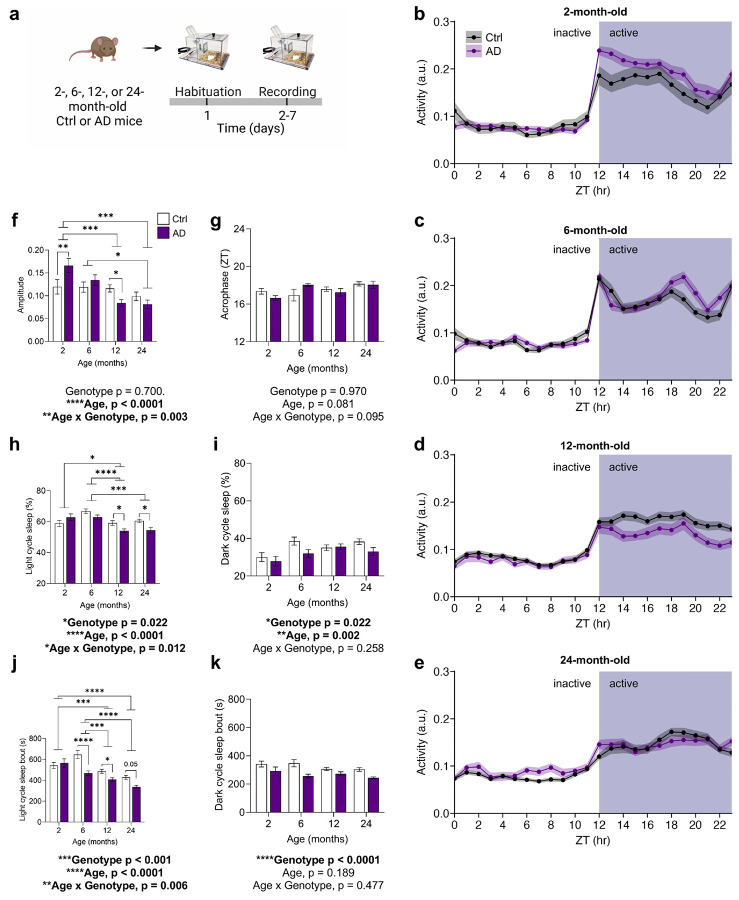
Steeper degradation of sleep-wake rhythms with aging in AD mice. **(a)** Experimental design for sleep-wake rhythm phenotyping with the PiezoSleep system in 2-, 6-, 12-, and 24-month-old Ctrl or AD mice. **(b-e)** Sleep-wake activity rhythms averaged across days for each zeitgeber hour (ZT0 aligned to light cycle onset) in 2-, 6-, 12-, and 24-month-old Ctrl or AD mice. Shaded ribbons represent the SEM. **(f)** Amplitude (oscillatory strength) of sleep-wake rhythms across age and genotype groups. **(g)** The acrophrase of sleep-wake rhythms (time to peak of rhythm relative to ZT0) across age and genotype groups. **(h, i)** Percentage of time spent asleep in the light (**h**, inactive) period and dark (**i**, active) period across age and genotype groups. **(j, k)** Average sleep bout length in the light (**j**) and dark (**k**) periods across age and genotype groups. Data shown as mean ± SEM (n = 12-29 per group, n = 5-14 female mice per group). *p < 0.05, **p < 0.01, ****p < 0.0001. Assessed using a two-way ANOVA with Tukey’s multiple comparison test (**f-k**).

Sleep–wake and sleep architecture disturbances are common and likely contribute to sundowning in dementia^26^. To model these features, we recorded sleep–wake activity in 2-, 6-, 12-, and 24-month-old control (Ctrl) and APPswe/PS1ΔE9 (AD) mice^23^ using the PiezoSleep system^27,28^ (Extended Data Fig. 1a). Sleep–wake rhythmicity weakened with age and declined prominently in AD (Extended Data Fig. 1b-e). Sinusoidal regression^29^ of activity revealed steeper age-dependent decline in rhythm amplitude (i.e. strength) in AD mice, partly reflecting early hyperactivity in 2-month-old AD mice during the dark (active phase (Extended Data Fig. 1b,f), which has been previously described^30^. By 12 months of age, AD amplitude fell below that of Ctrl mice. During the light (inactive) period, older AD mice showed reduced sleep time and shorter bouts (Extended Data Fig. 1h,j). These data support sleep-wake rhythm disruptions in AD mice beyond aging-related changes.

**Extended Data Fig. 2 ∣:**
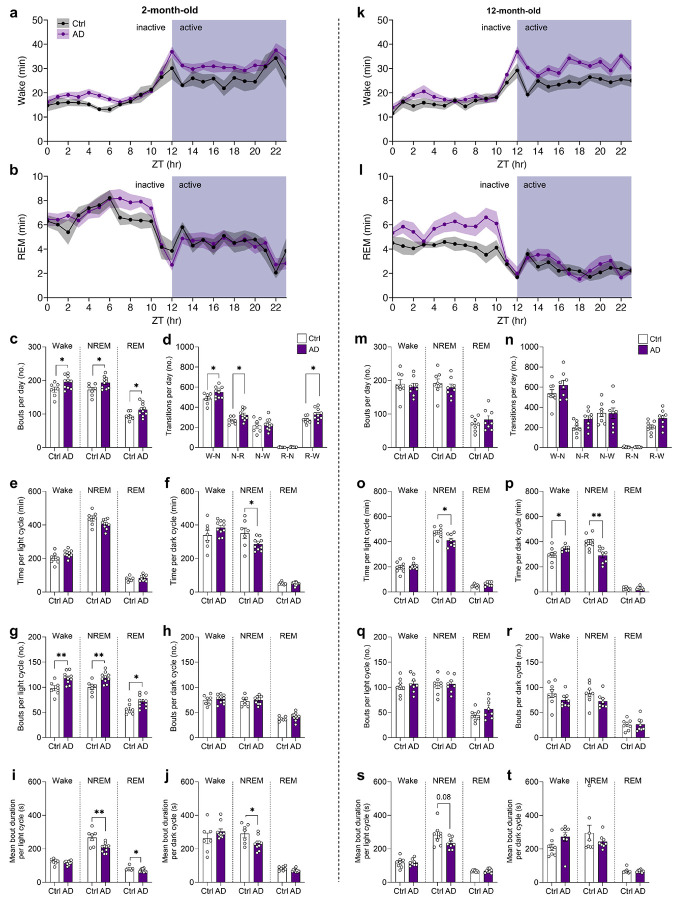
Early sleep fragmentation in young AD mice across the light and dark cycle. **(a, b)** Average time spent in (**a**) Wake and **(b)** REM sleep across each zeitgeber hour in 2-month-old Ctrl or AD mice (n = 7-10 per group; n = 3-5 female mice per group). **(c)** Higher number of bouts per day of Wake, NREM sleep or REM sleep in 2-month-old AD mice compared to Ctrl mice. **(d)** Counts of transitions **(**Wake to NREM, W-N; NREM to REM, N-R; NREM to Wake, N-W; REM to NREM, R-N; REM to Wake, R-W**)** between the states of Wake, NREM sleep, and REM sleep in 2-month-old mice. AD mice show higher number of W-N, N-R, and R-W transitions. **(e, f)** The average time spent in Wake, NREM or REM per (**e**) light or **(f)** dark cycle in 2-month-old mice. **(g, h)** The average number of bouts of Wake, NREM or REM per **(g)** light or **(h)** dark cycle in 2-month-old mice. AD mice show a higher number of bouts during the light cycle. **(i, j)** The average bout duration of Wake, NREM or REM per (**g**) light or (**h**) dark cycle in 2-month-old mice. **(k, l)** Average time spent in (**k**) Wake and (**l**) REM sleep across each zeitgeber hour in 12-month-old Ctrl or AD mice (n = 8 per group; n = 5 female mice per group). **(m)** Bouts per day of Wake, NREM or REM in 12-month-old mice. (**n)** Counts of all possible transitions between the states of Wake, NREM, and REM in 12-month-old mice. **(o, p)** The average time spent in Wake, REM or NREM per (**o**) light or (**p**) dark cycle in 12-month-old mice. **(q, r)** The average number of bouts of Wake, NREM or REM per (**q**) light or (**r**) dark cycle in 12-month-old mice. **(s, t)** The average bout duration of Wake, NREM or REM per (**s**) light or (**t**) dark cycle in 12-month-old mice. Data shown as mean ± SEM. *p < 0.05, **p < 0.01. Assessed using unpaired two-tailed t-tests (**c-j, m-t**).

**Extended Data Fig. 3 ∣:**
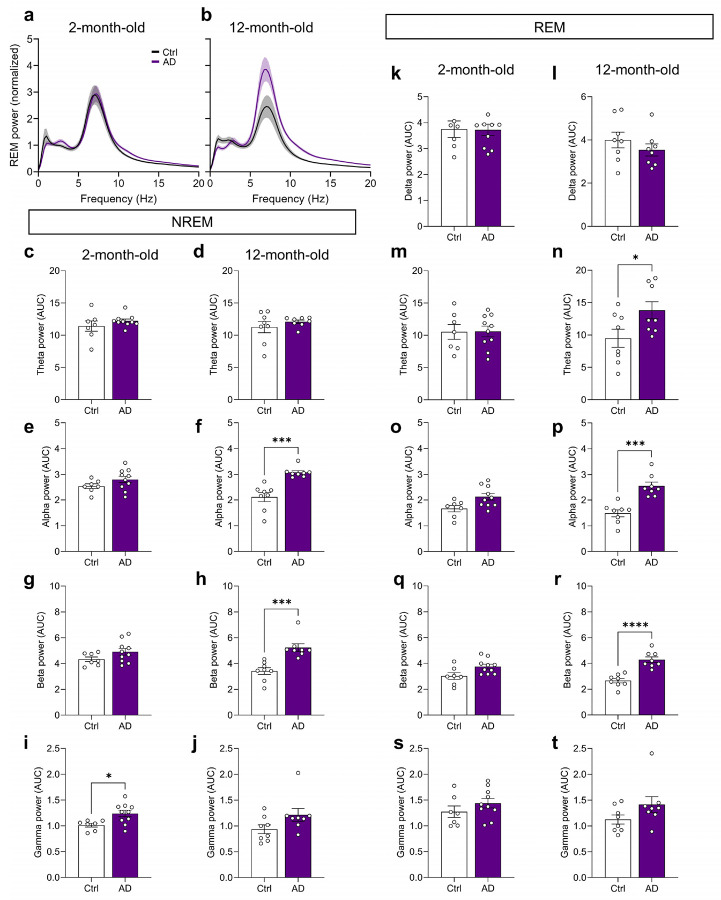
Altered sleep-stage dependent power spectra in aged AD mice. **(a, b)** Normalized EEG power spectra of **(a)** 2- (n = 7-10 per group; n = 3-5 female mice per group) or **(b)** 12-month-old Ctrl and AD mice (n = 8 per group; n = 5 female mice per group) during REM sleep. **(c, d)** Theta band power (4-10 Hz) in **(c)** 2- or **(d)** 12-month-old mice during NREM sleep. Band powers are expressed as the area under the curve (AUC) for each respective frequency range. See Fig. 1 for delta range comparison during NREM sleep in 2- or 12-month-old mice. **(e, f)** Alpha band power (10-13 Hz) in **(e)** 2- or **(f)** 12-month-old mice during NREM sleep. **(g, h)** Beta band power (13.1-30 Hz) in **(g)** 2- or **(h)** 12-month-old mice during NREM sleep. **(i, j)** Gamma band power (30.1-50 Hz) in **(i)** 2- or **(j)** 12-month-old mice during NREM sleep. **(k, l)** Delta band power (0.1-4 Hz) in **(k)** 2- or **(l)** 12-month-old mice during REM sleep. **(m, n)** Theta band power in **(m)** 2- or **(n)** 12-month-old mice during REM sleep. **(o, p)** Alpha band power in **(o)** 2- or **(p)** 12-month-old mice during REM sleep. **(q, r)** Beta band power in **(q)** 2- or **(r)** 12-month-old mice during REM sleep. **(s, t)** Gamma band power in **(s)** 2- or **(t)** 12-month-old mice during REM sleep. Data shown as mean ± SEM. *p < 0.05, ***p < 0.001, ****p < 0.0001. Assessed using unpaired two-tailed t-tests (**c-t**).

**Fig. 1 ∣:**
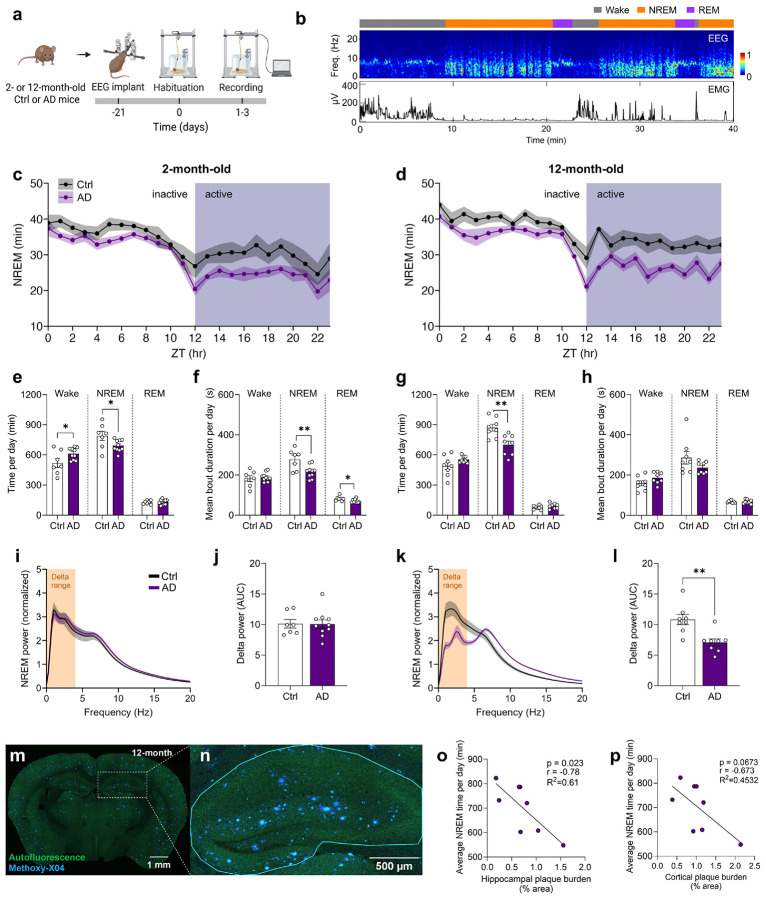
AD mice show sleep fragmentation and disrupted slow wave activity. **(a)** Experimental design for sleep architecture phenotyping. **(b)** Representative EEG/EMG recording (top bar, gray = Wake, orange = NREM sleep, purple = REM sleep; middle bar, EEG power spectrogram, 0–25 Hz; bottom bar, EMG traces). Average time spent in NREM sleep across zeitgeber hour (ZT0 aligned to light cycle onset) in **(c)** 2-month-old mice (n = 7-10 per group; n = 3-5 female mice per group) and **(d)** 12-month-old mice (n = 8 per group; n = 5 female mice per group). **(e,f)** The average time per day or average bout duration of wake, NREM sleep, and REM sleep in 2-month-old Ctrl or AD mice. Average time and bout duration of NREM sleep is decreased in AD mice. **(g,h)** The average time per day or average bout duration of wake, NREM sleep, and REM sleep in 12-month-old Ctrl or AD mice. The average time per day in NREM sleep is decreased in AD mice. **(i)** Normalized EEG power spectra of 2-month-old Ctrl and AD mice during NREM sleep. The orange shaded region represents the delta range (0.1-4 Hz). **(j)** Delta power during NREM sleep in 2-month-old mice. Power is expressed as the area under the curve (AUC) across the delta range. **(k)** Power spectra of 12-month-old Ctrl and AD mice during NREM sleep. **(l)** Delta power is reduced in 12-month-old AD mice. **(m)** Representative image of Aβ-plaques stained with Methoxy-X04 in a 12-month-old AD mouse. **(n)** Inset of the hippocampal region outlined in **m**; the blue line represents the boundary of the hippocampus. **(o,p)** Negative correlation between **(o)** hippocampal (HPC) or **(p)** cortical (CTX) plaque burden (% area) and total average time spent in NREM sleep across the 24 h cycle. Data shown as mean ± SEM. *p < 0.05, **p < 0.01. Assessed using unpaired *t*-tests (**e-h, j, l**). Assessed using linear regression (**o, p**).

Sleep architecture deficits are reported in AD, with Aβ and tau accumulation linked to disrupted NREM (“deep”) sleep, characterized by SWA in the delta range (0.1-4 Hz)^10,11^. We investigated sleep architecture by performing electroencephalography/electromyography (EEG/EMG) recordings in 2- and 12-month-old mice (Fig. 1a,b). At 2 months of age, AD mice spent less total time in NREM sleep per day, and exhibited sleep fragmentation, reflected by more time in wake, shorter NREM/REM bouts, more bouts overall, and increased state transitions (Fig. 1c-f and Extended Data Fig. 2). At 12 months of age, AD mice showed no changes in bout length, but had sustained decrease of NREM sleep time (Fig. 1g-h). We further investigated the power spectra during NREM sleep, as higher delta power is considered an indicator of SWA depth. During NREM sleep, EEG spectra were largely unchanged at 2 months of age (Fig. 1i,j; Extended Data Fig. 3). However, delta power was reduced in 12-month-old AD mice when compared with Ctrl mice (Fig. 1k,l). At higher frequencies, 12-month-old AD mice showed a rightward shift in the power spectrum during NREM sleep (Fig. 1k), reflected as heightened power in the alpha and beta ranges (Extended Data Fig. 3f-h).

Finally, we examined whether pathology burden correlated to NREM disruption by quantifying Aβ plaque burden in the hippocampus and cortex (Fig. 1m,n; Supplementary Fig. 1a-c). Delta power did not correlate with plaque burden (Supplementary Fig. 1d,e), but NREM sleep time negatively correlated with hippocampal, and to a lesser extent cortical, plaque load (Fig. 1o,p). Overall, the sleep architecture alterations in AD mice parallel the NREM sleep deficits reported in AD patients.

### Aged AD mice exhibit hyperactivity and aberrant exploration at Sundown but not Sunrise

**Extended Data Fig. 4 ∣:**
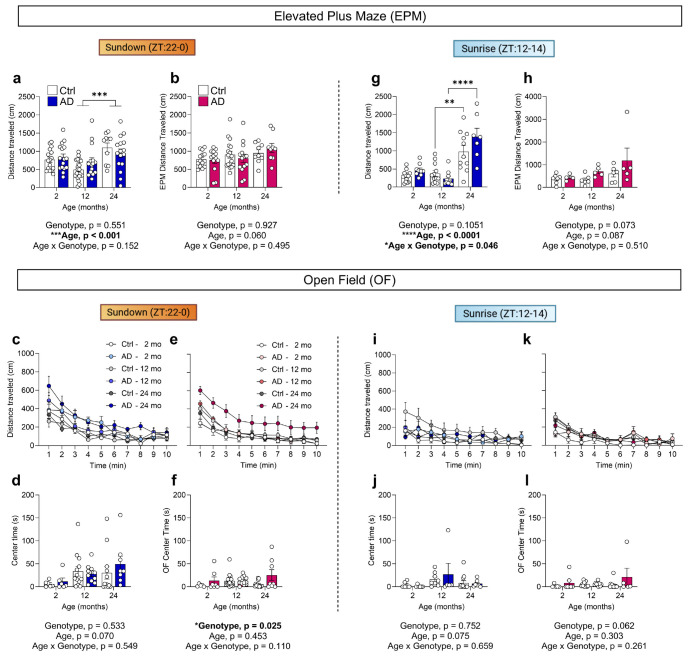
No alterations in anxiety-like measures in the open field in aged AD mice. **(a,b)** Total distance traveled in the EPM in **(a)** male and **(b)** female mice at Sundown. **(c)** Minute-by-minute distance traveled across 10 min of the OF assay in 2-, 12-, and 24-month-old Ctrl and AD male mice at Sundown. **(d)** The time spent in the center of the OF across age and genotype groups in male mice at Sundown. **(e)** Minute-by-minute distance traveled in the OF in female mice at Sundown. **(f)** The time spent in the center of the OF in female mice at Sundown. **(g,h)** Total distance traveled in the EPM in **(g)** male and **(h)** female mice retested at Sunrise. **(i)** Minute-by-minute distance traveled in the OF in male mice at Sunrise. **(j)** The time spent in the center of the OF in male mice at Sunrise. **(k)** Minute-by-minute distance traveled in the OF in female mice at Sunrise. . **(l)** The time spent in the center of the OF in female mice at Sunrise. Data shown as mean ± SEM (n = 5-21 per group). **p < 0.01, ****p < 0.0001. Assessed using a two-way ANOVA with Tukey’s multiple comparison test **(a, b, d, f, g, h, j, i)**.

**Fig. 2 ∣:**
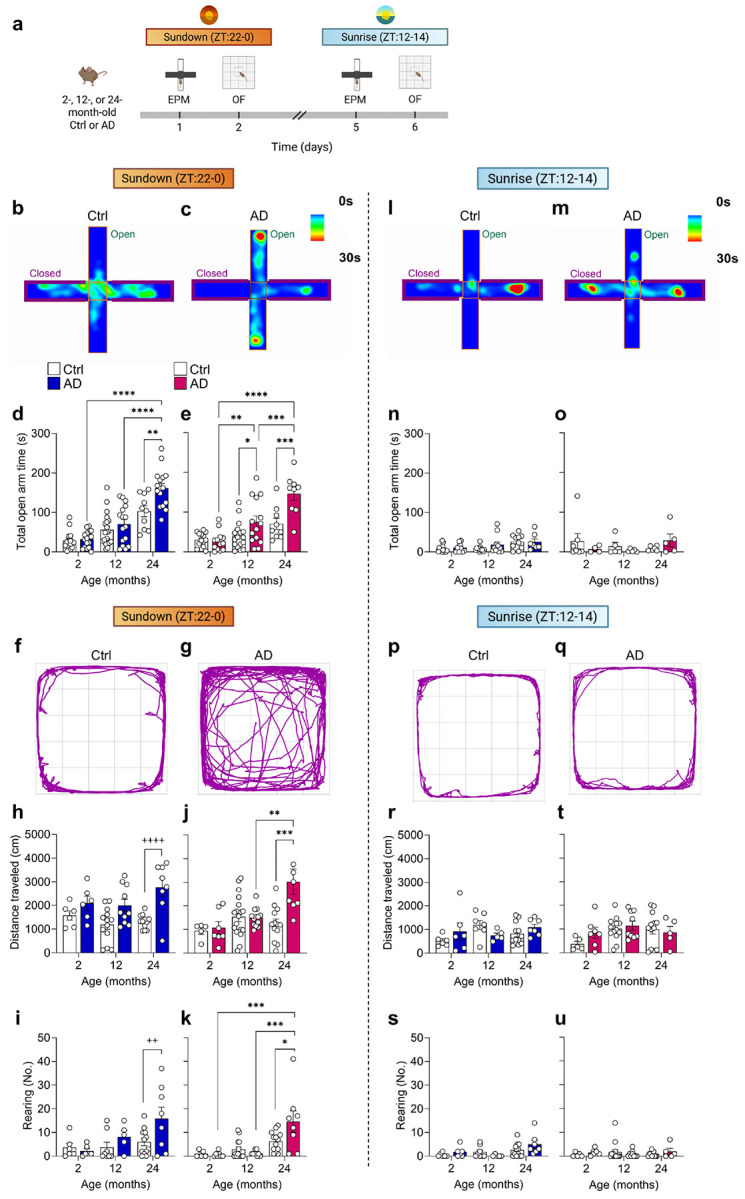
Aged AD mice exhibit aberrant exploration and hyperactivity in at Sundown. **(a)** Experimental design. Sundown is operationally defined as the last two hours of the active period (ZT22-0), whereas Sunrise is defined as the first two hours of the active period (ZT12-14). **(b-c)** Representative heatmaps of exploration in the EPM in 12-month-old female Ctrl or AD mice at Sundown. **(d,e)** Total time spent in the open arms of the EPM in 2-, 12-, and 24-month-old Ctrl and AD **(d)** male and **(e)** female mice at Sundown. Male 24-month-old AD mice explore the open arms more than male Ctrl mice, whereas female 12- and 24-month-old AD mice show higher open arm exploration than female Ctrl mice. **(f,g)** Representative OF tracking plots from 24-month-old male Ctrl or AD mice at Sundown. **(h,i)** Distance traveled and number of rears in 2-, 12-, and 24-month-old male in the OF at Sundown**. (j,k)** Distance traveled and number of rears in female mice at Sundown. **(l,m)** Representative EPM heatmaps in 12-month-old female Ctrl or AD mice at Sunrise. **(n,o)** Time in the open arms of the EPM in **(n)** male and **(o)** female mice at Sunrise. **(p,q)** Representative OF tracking plots from 24-month-old male mice at Sunrise. **(r,s)** Distance traveled and number of rears across all age and genotype groups in male mice at Sunrise. (**t,u)** Distance traveled and number of rears across all age and genotype groups in male mice at Sunrise. Data shown as mean ± SEM (n = 5-21 per group). *p < 0.05, **p or ^++^p < 0.01, ***p < 0.001, ****p or ^++++^p < 0.0001. Assessed using a two-way ANOVA with Tukey’s multiple comparison test **(d-e, h-k, n-o, r-u)**. ^+^, represents comparisons conducted based on a priori interest and trending interaction effects (**h, i**).

Sundowning-like behaviors were investigated using the open-field (OF) and elevated plus maze (EPM) assays, which better assess exploration and affective state in rodents than locomotor averages^31^ (Fig. 2). Both assays allow for unconstrained behavior and possess zones considered anxiogenic (center of the OF and open arms of the EPM)^31,32^. Sunrise (zeitgeber hour, ZT12-14) and Sundown (ZT22-0) were defined as the first and last 2 hours of the active period, respectively. Two-, 12-, and 24-month-old Ctrl and AD mice were assayed in the EPM and OF on consecutive days at Sundown and retested 5 days later at Sunrise (Fig. 2a; Extended Data Fig. 4).

At Sundown, 24- but not 2- or 12-month-old male AD mice showed increased open arm exploration in the EPM, whereas 12- and 24-month-old female AD mice both exhibited increased open arm exploration, indicating earlier behavioral onset than male mice (Fig. 2b-e). Since open arm exploration did not align with increased OF center time in aged AD mice (Extended Data Fig 4d,f), rather than decreased anxiety, in the context of AD pathology we considered this behavior a potential type of aberrant exploration. In the OF, 24- but not 2- or 12-month-old male AD mice exhibited increases in distanced traveled and rearing (Fig. 2f-i), with rearing indicating exploratory or potential escape behavior^33^. Similarly, 24- but not 2- or 12-month-old female AD mice exhibited increased distance traveled and rearing (Fig. 2j,k). At Sunrise, all groups spent a comparable amount of time in the EPM open arms (Fig. 2l-o), and exhibited similar distances traveled and rearing levels in the OF (Fig. 2p-u). Together, these results highlight hyperactivity and aberrant exploration in male and female AD mice at Sundown but not Sunrise. While hyperactivity has been reported in other APP/PS1 mouse models^30,34^, to our knowledge, this is the first report of a systematic time-of-day-dependent hyperactivity in the OF.

**Extended Data Fig. 5 ∣:**
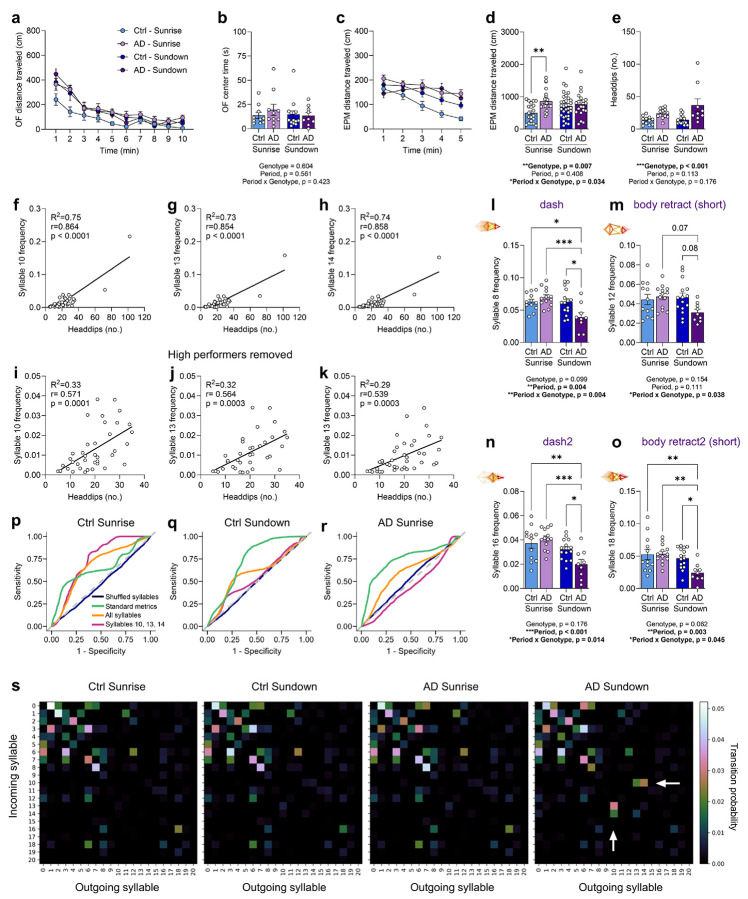
KPMS syllables correlate with head dipping behavior and transition matrices reveal a dynamic behavioral signature. **(a)** Minute-by-minute distance traveled across 10 min of the OF during first-time exposure to the OF in 12-month-old female Ctrl and AD mice at Sunrise or Sundown. **(b)** No difference in total time spent in the center of the OF across all Ctrl and AD groups at Sundown or Sunrise. **(c)** Minute-by-minute distance traveled across 5 min of the EPM across all groups. **(d)** Total distance traveled in the EPM across all groups. **(e)** Total number of head dips in the EPM (assessed as number of nose-point crossing across the bordering edges of each open arm) across all groups. **(f-h)** Frequency of KPMS syllables 10, 13, and 14 correlated with the number of head dips measured across all mice. **(i-k)** As in **f-h**, but with two mice with especially high head-dipping performance removed. Strong correlation is retained even without high performers. **(l-o)** The frequencies of syllables 8, 12, 16, and 18 across all groups. Purple headings are experimenter-given descriptors of the type of movement represented by the syllables. **(p-r)** An ROC curve for the Ctrl Sunrise, Ctrl Sundown, and AD Sunrise classes, summarizing logistic regression model performance per class. Curves are color-coded by the predictor used. **(s)** Syllable transition matrices, where the probabilities of all possible syllable transitions (Incoming syllable) given a current syllable state (Outgoing syllable) is indicated by color. White arrows reveal a dynamic signature in the AD Sundown group showing higher probabilities of transitioning to syllables 13 and 14, given a current state of syllable 10 or a higher probability of transitioning to syllable 10, given a current state of syllable 13 or 14. Data shown as mean ± SEM (n = 9-28 per group). *p < 0.05, **p < 0.01, ***p < 0.001. Assessed using a two-way ANOVA with Tukey’s multiple comparison test (**b, d, e, l-o**). Assessed using linear regression (**f-k**).

**Fig. 3 ∣:**
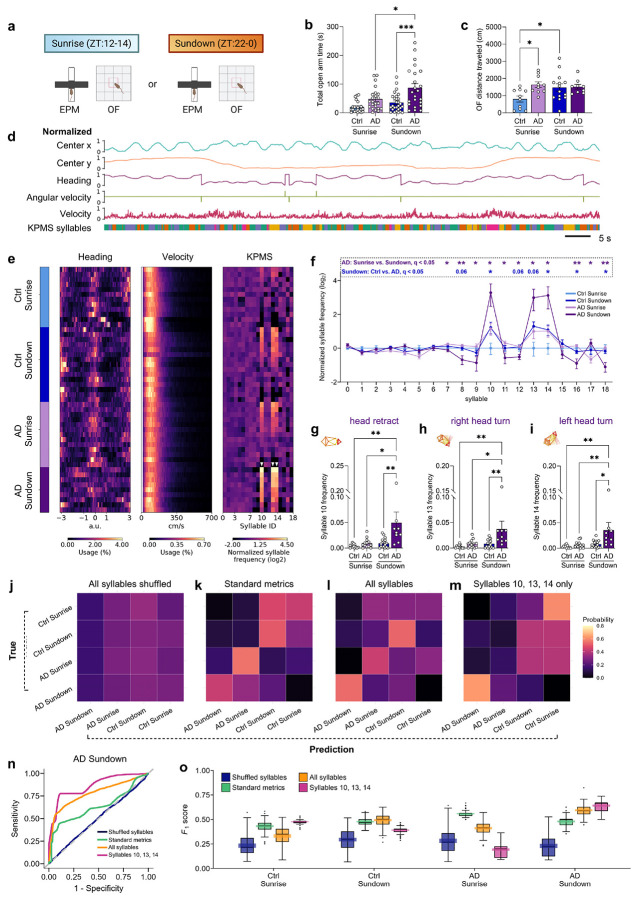
Unsupervised pose estimation reveals a unique behavior signature of AD mice at Sundown. **(a)** Behavioral design comparing first-exposure performance in OF and EPM in 12-month-old Ctrl or AD female mice at Sundown and Sunrise. **(b)** Time spent in the open arms of the EPM in Ctrl or AD mice at Sundown or Sunrise. **(c)** Distanced traveled in the OF in Ctrl or AD mice at Sundown or Sunrise. **(d)** Representative 30 s behavior trace of behavior metrics during EPM testing, including centroid x- and y- position, heading (orientation), angular velocity, and velocity. Values are normalized across each metric’s dynamic range. Bottom bar indicates distinct color-coded behavioral syllables categorized by Keypoint-MoSeq (KPMS). **(e)** Behavioral summary of each mouse’s performance in the EPM. Class assignments (period and genotype subgroup) per mouse are indicated as a color-coded bar (left). The distribution of heading (−3-3, arbitrary units, a.u.) and velocity values (0-700 cm/s) based on frequency of usage are shown on the left and middle columns. The rightmost column indicates the normalized expression frequency of each KPMS syllable (log2 transformed after normalization by the mean syllable frequency of the Ctrl Sunrise group). **(f)** Averaged normalized syllable frequency. Color-coded asterisks indicate the FDR-adjusted significant q values between either AD Sunrise and AD Sundown comparisons or between Ctrl Sundown and AD Sundown comparisons. **(g-i)** Targeted comparisons of frequencies (unnormalized) of KPMS syllables 10, 13, and 14. Purple headings are experimenter-given descriptors of the observed movement represented by the syllables. **(j-m)** Classification matrices (rows = true class, columns = predicted class), summarizing performance of a linear classifier after 200 repeat 8-fold cross-validations (see Methods). Predictors used were either shuffled **(j)** KPMS syllable frequences, **(k)** standard EPM metrics, **(l)** all KPMS syllable frequencies or **(m)** KPMS syllables 10, 13, and 14 only. Probability values were calculated by dividing by the true class total (row sums). **(n)** An ROC curve showing the true positive rate (sensitivity) plotted against the false positive rate (1-specificity) for the AD Sundown class. Curves are color-coded by the predictor used. **(o)** Boxplots of F_1_ scores summarizing classification performance for each class per predictor type. Upper and lower bounds of transparent boxes overlaying each boxplot represent the 99% confidence intervals. Data shown as mean ± SEM (n = 9-28 per group). *p or q < 0.05, ** p or q < 0.01, ***p < 0.001. Assessed using a two-way ANOVA and Sidak’s multiple comparison test (**b, c**) or Tukey’s multiple comparison test (**g, h, i**). Assessed with a three-way-mixed ANOVA with FDR-adjusted multiple Mann–Whitney U-tests (**f**). Individual comparisons were conducted based on *a priori* interest and trends observed (**b**).

To assess if our results were confounded by repeated testing, we compared first-time performance in the OF and EPM at Sunrise and Sundown of 12-month-old female mice, the youngest group showing aberrant exploration in the EPM (Fig. 3a; Extended Data Fig. 5). In the EPM, we confirmed that AD female mice at Sundown spend more time exploring the open arms compared to Ctrl female mice—an effect absent at Sunrise (Fig. 3b). In the OF, we confirmed that Ctrl and AD mice exhibited similar distances traveled; however, female AD mice exhibited increased distance traveled when compared to Ctrl mice at Sunrise (Fig. 3c). These data indicate that testing order does not impact performance in the EPM.

Lastly, we hypothesized that the atypical antipsychotic brexpiprazole, the only FDA-approved drug treating agitation in AD^35,36^ would impact sundowning-like behavior in AD mice. Mice were administered vehicle or brexpiprazole 1 h before the OF or EPM (Supplementary Fig. 2). At Sundown, brexpiprazole decreased distance traveled in the OF and decreased time in spent in the open arms of the EPM, indicating brexpiprazole may be an effective symptomatic treatment for sundowning.

### Unsupervised pose estimation reveals a stereotyped behavior signature of AD mice at Sundown

More qualitative observation revealed that aged AD mice at Sundown exhibit a striking stereotyped and repetitive way of exploring the open arms that differed from that of Ctrl mice (Supplementary Videos 1-2). We observed recurring head turning, head dipping, and pressured movement. To better characterize this behavior in the EPM, we leveraged computational ethology tools such as Social LEAP Estimates Animal Poses (SLEAP) for frame-by-frame pose-tracking^37^ and Keypoint-MoSeq^38^ (KPMS), which allows for unsupervised detection of behavioral structure organized into “syllables”^39,40^. This approach avoids limiting analyses to predefined behaviors and is egocentric rather than allocentric, meaning body position relative to the apparatus is not explicitly analyzed.

Using SLEAP and KPMS, we extracted syllable classifications from movement data across video recordings (Fig. 3d) and plotted behavioral summaries across mice (Fig. 3e). While typical measures like heading (directionality) or velocity did not distinguish groups, there was a prominent increase in frequency of KPMS syllables 10, 13, and 14 in the AD Sundown group and, to a lesser extent, in the Ctrl Sundown and AD Sunrise groups (Fig. 3e-f). These syllables resembled head retraction (Fig. 3g), right head turning (Fig. 3h), and left head turning (Fig. 3i, Supplementary Videos 1, 3–6;) and correlated with head-dipping behavior (Extended Data Fig. 5). Subtler changes also distinguished the AD Sundown group, including decreased frequencies of syllables 8, 12, 16, and 18 (Fig. 3f; Extended Data Fig. 5l-o), which we hypothesized was the behavioral expense of overperformance of syllables 10, 13, and 14. Transition probabilities amongst syllables 10, 13, and 14 were also highest in the AD Sundown group (Extended Data Fig. 5).

We next assessed if KPMS syllables contained information more useful for predicting the AD Sundown class compared to typical metrics. We trained linear classifiers using shuffled syllables, standard behavioral metrics, all KPMS syllables, or just syllables 10, 13, and 14 as predictors (Fig. 3j-m). ROC curves for the AD Sundown class showed syllables 10, 13, and 14 performed best as predictors, although used of all syllables performed second-best (Fig. 3n). We examined F1 scores (e.g., a composite metric on ability to detect true positives and reject false positives and negatives) and confirmed that use of syllables 10, 13, and 14 alone, followed closely by use of all syllables, resulted in the best model performance for the AD Sundown class (Fig. 3o). Interestingly, syllables 10, 13, and 14 seemed to underperform against standard metrics for the Ctrl Sundown and AD Sunrise classes, likely due to similar expression of these syllables between these groups. Overall, a stereotyped turning motion associated with head dipping remained the most distinctive feature of the AD Sundown group, which demonstrates KPMS syllables contain additional useful behavioral features beyond that captured by standard behavioral metrics.

### Disrupted time-of-day-dependent activation of vasopressin cells in the SCN of AD mice

**Fig. 4 ∣:**
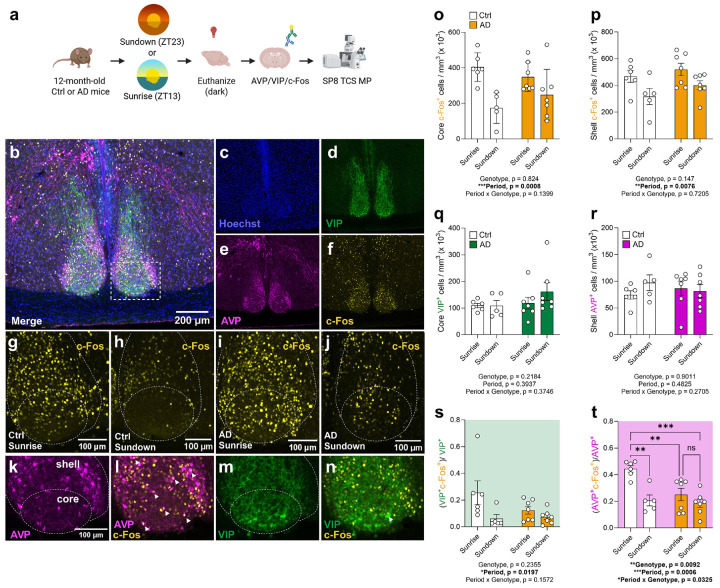
Disruption of time-of-day-dependent cell type-specific activation in the suprachiasmatic nucleus of AD mice. **(a)** Experimental design for co-staining vasopressin (AVP), vasoactive-intestinal peptide (VIP), and c-Fos expression in Ctrl and AD mice in the suprachiasmatic nucleus (SCN). **(b-f)** Representative **(b)** co-staining of cell nuclei with (**c)** Hoechst in (**d**) VIP^+^-, **(e)** AVP^+^-, and **(f)** c-Fos^+^-expressing cells. **(g-j)** Representative images of c-Fos expression in the SCN from mice in the Ctrl Sunrise, Ctrl Sundown, AD Sunrise, and AD Sundown groups. The core and shell subdivisions are outlined by white dotted lines. **(k-n)** A zoomed inset of the outlined region in **b** (white dashed lines) highlighting **(k)** AVP and **(m)** VIP expression in the SCN core and shell, as well as co-expression with (**l**, **n**) c-Fos^+^ cells. Identified co-labelled cells are denoted by white arrows. **(o-p)** c-Fos^+^ cell counts in the **(o)** core and **(p)** shell subdivisions of Ctrl and AD mice at Sunrise and Sundown. **(q)** VIP^+^ cell counts in the core of the SCN across groups. **(r)** AVP^+^ cell counts in the shell of the SCN across groups. **(s)** Number of cells colabelled with VIP^+^ and c-Fos^+^ normalized by total VIP^+^ cell counts (activated proportion of the VIP^+^ population). **(t)** Colabelled AVP^+^ and c-Fos^+^ cells normalized by total AVP^+^ cell counts (activated proportion of the AVP^+^ population). (n = 5-7 mice per group). *p < 0.05, **p < 0.01, ***p < 0.001. Error bars represent ± SEM. Assessed using a two-way ANOVA with Tukey’s multiple comparison test (**o-t**).

We hypothesized that some of the behavioral alterations observed may be related to dysregulation of circadian circuitry. To investigate changes in circadian function, we analyzed subdivisions of the SCN across time of day. The SCN is topographically organized into a dorsal shell region, composed of primarily vasopressin (AVP)- expressing cells, and a ventral core region composed of vasoactive intestinal peptide (VIP)-expressing cells^41,42^. Activity in the SCN has been shown to be topographically organized and oscillate differentially across the day in its different subdivisions^43,44^. We posited there may be time-of-day differences in SCN core and shell activation. Brains from 12-month-old female Ctrl and AD mice—the youngest group exhibiting aberrant behavior—were extracted at Sundown or Sunrise (Fig. 4a). Afterwards, AVP, VIP, and c-Fos expression was quantified in the SCN (Fig. 4b-n). c-Fos^+^ expression was decreased at Sundown, with no effect of Genotype (Fig. 4o,p), consistent with expected diurnal oscillation of activity. VIP^+^ and AVP^+^ expression were not impacted by Genotype or Period (Fig. 4q,r). The activated proportion of VIP^+^ cells (VIP^+^c-Fos^+^/VIP^+^) was decreased at Sundown, with no effect of Genotype (Fig. 4s). In contrast, in Ctrl mice, the activated proportion of AVP^+^ cells (AVP^+^c-Fos^+^/AVP^+^), showed an expected decrease at Sundown, whereas this diurnal pattern was blunted in AD mice (Fig. 4t). These data indicate disrupted day-night rhythmicity of AVP neurons in the SCN of AD mice.

### Hyperconnectivity amongst sensorimotor and cortical regions in AD functional networks at Sundown

**Fig. 5 ∣:**
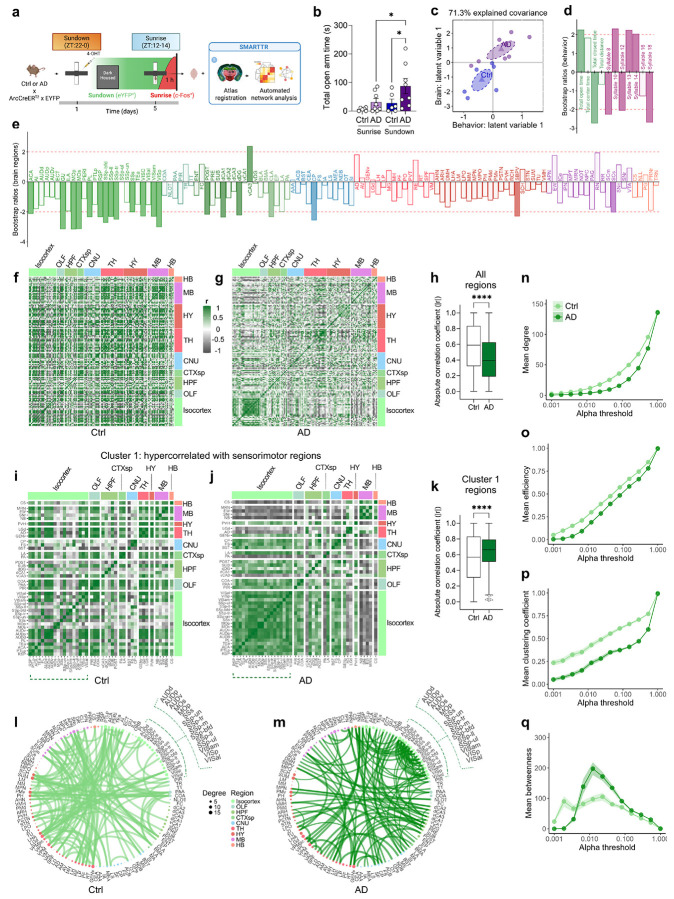
A sensorimotor hyperconnectivity signature found in AD functional networks at Sundown time. **(a)** Experimental design for tagging neural activity during the EPM at Sundown and Sunrise in 12-month-old female Ctrl or AD ArcCreER^T2^ x eYFP mice. **(b)** Time spent in the open arms of the EPM in at Sundown and Sunrise in Ctrl and AD mice. **(c)** The first latent dimension space following behavior partial least squares correlation analysis (PLSC) for behavioral and brain-activation measures at Sundown. Plotted circles are color-coded by genotype and represent values from individual mice within the latent space. Triangles denote the group mean and shaded polygons bounded by dotted lines represent the bootstrapped 95% confidence intervals. **(d)** Bootstrap ratios (BR) denoting the original behavioral features that reliably contribute to the first behavioral latent variable. A BR of ± 2 corresponds to a 95% confidence interval cut-off. Standard EPM metrics are outlined in green, whereas Keypoint-MoSeq (KPMS) syllables 8, 10, 12, 13, 14, 16, 18 are outlined in pink. Features with a ∣BR∣ > 2 have color-filled bars. **(e)** BRs for the regional activity (eYFP^+^ cells / mm^3^) contributing most reliably to the first brain latent variable. Regions are sorted and color-coded based on their corresponding super regions: Isocortex; olfactory bulb, OLF; hippocampal formation, HPF; cortical subplate, CTXsp; cerebral nuclei, CNU; thalamus, TH; hypothalamus, HY; midbrain, MB; hindbrain, HB. **(f, g)** Brain-wide region correlation heatmaps of eYFP^+^ activity across all mapped regions (137) for **(f)** Ctrl and **(g)** AD groups. **(h)** Distributions of absolute Pearson’s r values across all regional correlations in Ctrl and AD groups. **(i, j)** Region correlation heatmaps for Ctrl **(i)** and AD **(j)** groups of a subgroup of regions (Cluster 1) identified by hierarchical clustering of brain-wide AD correlation patterns. Cluster 1 regions show high correlated activity with and amongst sensorimotor regions. **(k)** Absolute Pearson’s r values amongst Cluster 1 regions in Ctrl and AD groups. (**l, m)** Representative networks of Ctrl and AD groups highlighting the most significant correlations (edges are proportionally thresholded to 1.5% edge density by p-value). Sensorimotor regions labels are magnified and indicated by dotted green lines. (**n-q**) Trajectories of global topology metrics, including **(n)** mean degree, **(o)** efficiency**, (p)** clustering coefficient, and **(q)** betweenness centrality for Ctrl and AD networks across a range of alpha values for edge thresholding. Data shown as mean ± SEM (n = 6-8 per group). *p < 0.05, ****p < 0.0001. Assessed with a mixed ANOVA and uncorrected Fisher’s LSD (**b**). Unpaired two-tailed t-tests (**h, k**).

We next hypothesized that brain-wide activity patterns may be disrupted during sundowning-like behavior and utilized the ArcCreER^T2^ mice^25^ to identify activated neural patterns. Female 12-month-old Ctrl or AD x ArcCreER^T2^ x eYFP mice were tested in the EPM at Sundown (Fig. 5a). Immediately after, mice were injected with 4-hydroxytamoxifen (4-OHT) to tag activated neurons with eYFP^+^. Five days later, mice were retested at Sunrise and euthanized 1 h later to capture c-Fos expression. As previously shown, AD mice exhibited increased open-arm time at Sundown, but not at Sunrise (Fig. 5b). Sunrise and Sundown ensembles were then mapped and analyzed using the SMARTTR^24^ package for immediate early gene (IEG) mapping and network analysis (Supplementary Fig. 3). AD mice exhibited lower brain-wide eYFP^+^ expression at Sundown (Supplementary Fig. 4), which was not observed in brain-wide c-Fos^+^ expression at Sunrise (Supplementary Fig. 5).

**Extended Data Fig. 6 ∣:**
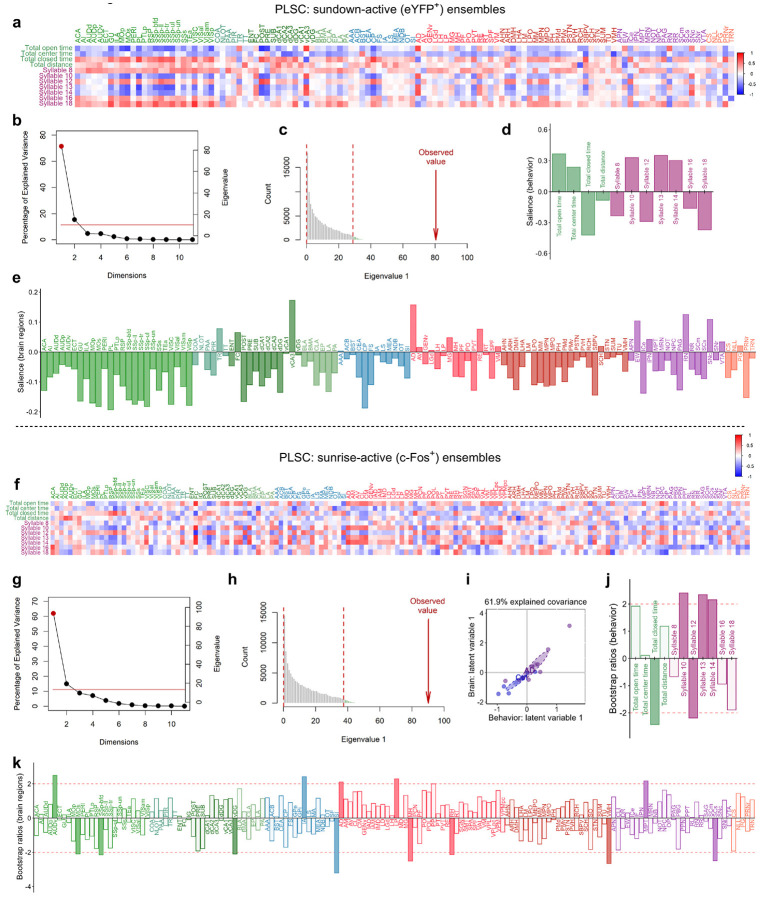
PLSC Latent variables capture structure of correlated behavior and brain activation patterns at Sundown and Sunrise. **(a)** Correlation heatmap between behavioral EPM metrics and brain activation (eYFP^+^ cells / mm^3^) across regions at sundown. Labels for standard EPM metrics are outlined in green, whereas KPMS syllables 8, 10, 12, 13, 14, 16, 18 are outlined in pink. Regions are sorted and color-coded based on their corresponding super regions: Isocortex; olfactory bulb, OLF; hippocampal formation, HPF; cortical subplate, CTXsp; cerebral nuclei, CNU; thalamus, TH; hypothalamus, HY; midbrain, MB; hindbrain, HB. **(b)** A scree plot showing the percentage of variance explained by each latent variable (LV) pair (i.e., dimension) following partial least squares correlation (PLSC). The red horizontal line represents the Kaiser criterion (average inertia or average variance per dimension). LVs statistically above chance after permutation testing are plotted with red dots (LV1, p = 0.0001). **(c)** A histogram showing the permuted null distribution for the first singular value (eigenvalue 1 or the covariance strength of LV1). Vertical dotted red lines indicate the bounds of the 95th percentile, and the red arrow indicates the observed eigenvalue. **(d)** Saliences representing the “weights” of the original behavioral features contributing to LV1 for behavior. **(e)** Saliences of the original brain activation features contributing to LV1 for brain activity. **(f)** Correlation heatmap between behavioral EPM metrics and brain activation (c-Fos^+^ cells / mm^3^) across regions at Sunrise. **(g)** Scree plot showing LVs statistically above chance (red dot, LV1, p = 0.001) after performing PLSC and permutation testing on behavior and brain activation data at Sunrise. **(h)** Permuted null distributions for the first singular value. **(i)** First latent dimension space (LV1) for behavioral and brain-activation measures at Sunrise. Plotted circles are color-coded by genotype and represent values from individual mice within the latent space. Triangles denote the group mean and shaded polygons bounded by dotted lines represent the bootstrapped 95% confidence intervals. **(j)** Bootstrap ratios (BR) denoting the original behavioral features that reliably contribute to LV1. A BR of ± 2 corresponds to a 95% confidence interval cut-off. Features with a ∣BR∣ > 2 have color-filled bars. **(k)** BRs for the brain region activity (c-Fos^+^ cells / mm^3^) contributing most reliably to LV1. Significance assessed using permutation analysis (**b, c, g, h,** see Methods).

Behavioral partial least squares correlation (PLSC)^45,46^ relates multivariate behavioral and brain activation patterns by identifying latent variable pairs—brain and behavioral components that maximize shared covariance in a lower-dimensional space. We applied PLSC to eYFP^+^ ensemble data and key behavioral metrics and found a significant brain–behavior relationship (fixed inertia = 145.65, *p* = 0.0007) (Extended Data Fig. 6). The first latent variable (LV1) captured 71.3% of shared variance (*p* = 0.0001; Extended Data Fig. 6a-e). Group projections onto LV1 (Fig. 5c) showed clear separation between AD and Ctrl mice at Sundown, with non-overlapping confidence intervals (CI) of group means. In contrast, Sunrise analyses yielded overlapping CI between groups in LV1 (Extended Data Fig. 6f-k). These findings indicate the brain-behavior relationship distinctly separates Ctrl and AD mice at Sundown but not Sunrise.

We next calculated bootstrap ratios (BRs)^47^, which are analogous to *z*-scores, to identify features most stably contributory to LV1. Behavioral features with ∣BR∣ > 2 included total open- and closed-arm time and KPMS syllables 8, 10, 12, 13, 14, and 18 (Fig. 5d). Unsurprisingly, time in the open arm and syllables 10, 13, 14 showed aligned BR directionality, indicating positive correlation. The brain regions most contributory to LV1 (Fig. 4e) could be categorized into functional groups: decision-making/attention (ACA; PL)^48^, sensorimotor processing (gustatory areas, GU; primary somatosensory area, SSp; supplemental somatosensory area, SSs; primary motor area, MOp; secondary motor area, MOs; visceral area, VISC; visual areas, VIS), navigational memory and contextual processing (retrosplenial area, RSP; dorsal CA1, dCA1; dorsal dentate gyrus, dDG; postsubiculum, POST), emotional regulation and anxiety processing (ACA; PL; ventral CA3, vCA3), motor control (caudoputamen, CP), and circadian processing (subparaventricular zone, SBPV)^41,48-60^.

**Extended Data Fig. 7 ∣:**
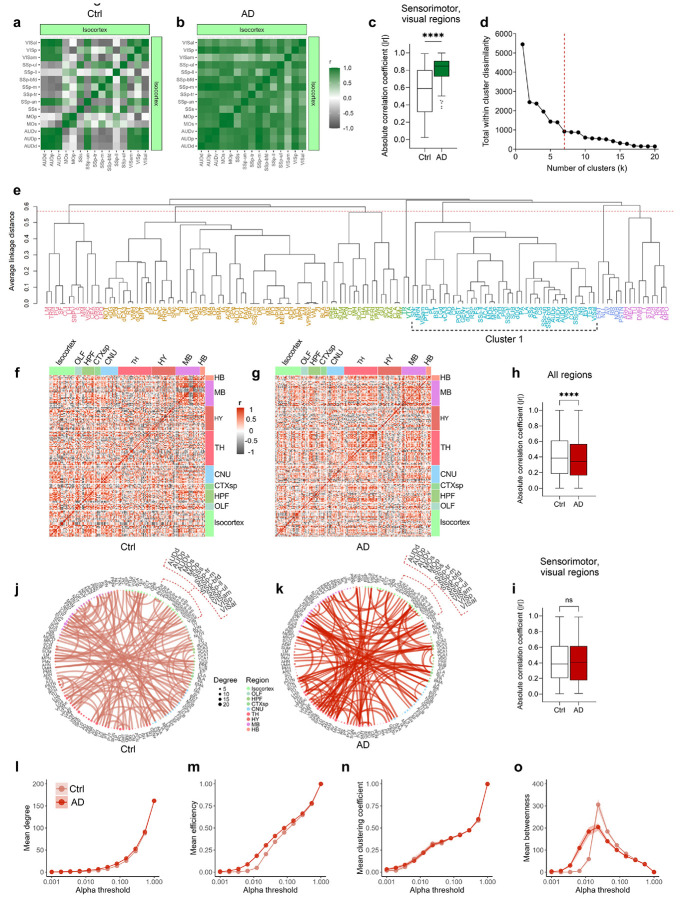
Heightened sensorimotor correlation at Sundown whereas similar functional connectivity patterns are not found at Sunrise. **(a, b)** Correlation heatmaps of Sundown-active (eYFP^+^ ensembles) activity across auditory, motor, somatosensory, and visual regions in **(a)** Ctrl or **(b)** AD ArcCreER^T2^ x eYFP mice. **(c)** Distributions of absolute Pearson’s r values in Ctrl or AD mice across sensorimotor regions shown in **a** and **b**. **(d)** The sum of within cluster distances for each k-number of clusters after hierarchical clustering of a brain-wide correlation patterns of AD mice at Sundown (eYFP^+^ ensembles). Using the “elbow” heuristic, k = 7 was chosen (vertical dotted line) due to diminishing reduction of total within cluster dissimilarity beyond this threshold. **(e)** A dendrogram showing the results of hierarchical clustering on AD eYFP^+^ regional correlations. A horizontal red dotted line denotes the tree cut height resulting in k = 7. Regions are color-coded by each cluster they belong to. Cluster 1 regions are regions that show especially high correlated activity with and amongst sensorimotor regions previously shown in **a, b**. **(f, g)** Brain-wide regional correlation heatmaps of c-Fos^+^ activity across all mapped regions for **(f)** Ctrl and **(g)** AD groups. **(h, i)** Distributions of absolute Pearson’s r values across **(h)** all regional correlations or restricted to **(i)** sensorimotor regions in Ctrl and AD groups. **(j, k)** Representative networks of Ctrl and AD groups highlighting the most significant correlations (edges are proportionally thresholded to 1.0% edge density by p-value). Sensorimotor regions labels are magnified and indicated by dotted red lines. (**l, m, n, o**) Trajectories of global topology metrics, including **(l)** mean degree, **(m)** efficiency, **(n)** clustering coefficient, and **(o)** betweenness centrality for Ctrl and AD networks across a range of alpha values for edge thresholding. Data shown as mean ± SEM (n = 6-8 per group). ****p < 0.0001. Unpaired two-tailed t-tests (**h, i**).

As co-activation or opposing activation can signify functional connectivity, we constructed heatmaps of correlated IEG expression at Sundown (Fig. 5f,g). Overall functional connectivity (∣Pearson’s r∣) was lower in AD than in Ctrl mice (Fig. 5h), yet a cluster of primarily sensorimotor isocortical regions, showed stronger intercorrelations in AD mice (Extended Data Fig. 7a-c). To identify all distributed regions associated with this signature, we performed hierarchical clustering on correlations from the AD group. One cluster, Cluster 1, encompassed all hyper-correlated sensorimotor regions (Extended Data Fig. 7d,e) alongside additional isocortical (ACA; RSP; PL; posterior parietal association areas, PTLp), olfactory (piriform area, PIR; piriform-amygdalar area, PAA; cortical amygdalar area, COA), hippocampal (vCA3, dCA1, dDG, POST), amygdalar/limbic (lateral amygdala, LA; posterior amygdala, PA; bed nucleus of the stria terminalis; BST), and striatal (CP; substantia nigra, reticular part, SNr) regions. Notably, most regions contributing to LV1 in the PLSC analysis overlapped with Cluster 1. Correlation heatmaps restricted to Cluster 1 revealed highly aligned or inversely coupled patterns among AD regions that was absent in the Ctrl group (Fig. 5i,j), with higher absolute correlation values in AD mice (Fig. 5k). Altogether, these findings indicate heightened sensory–motor integration and engagement of hippocampal, striatal, and limbic circuits involved in emotional and attentional processing in AD mice at Sundown, but not Sunrise.

We next constructed networks with all brain regions as nodes and correlations as edges (edge density = 1.5%) and confirmed dense sensorimotor hyperconnectivity in AD mice, that was absent in Ctrl mice (Fig. 5l,m) and at Sunrise (Extended Data Fig. 7j,k), Since threshold choice affects network properties^61,62^, we iteratively constructed networks across different alpha thresholds and calculated topological metrics: degree is the number of connections per node; the clustering coefficient reflects how likely a node’s neighbors are also connected, indicating functional specialization^63^. Efficiency measures how easily information travels across nodes, while betweenness quantifies how often a node lies on the shortest path between others, reflecting influence over information flow. Mean degree (Fig. 5n), efficiency (Fig. 5o), and clustering coefficient (Fig. 5p), were lower in AD networks; however, mean betweenness (Fig. 5q) increased and decreased more sharply in AD networks, suggesting a smaller subset of highly connected, influential nodes. Conversely, differences in sensorimotor hyperactivity and network topology patterns were not observed between Ctrl and AD mice at Sunrise (Extended Data Fig. 7f-o). Overall, these finding supports a global network-level disruption at Sundown but not Sunrise in AD mice.

### Salience network connectivity is contingent on time of day and cognitive status in human subjects

**Fig. 6 ∣:**
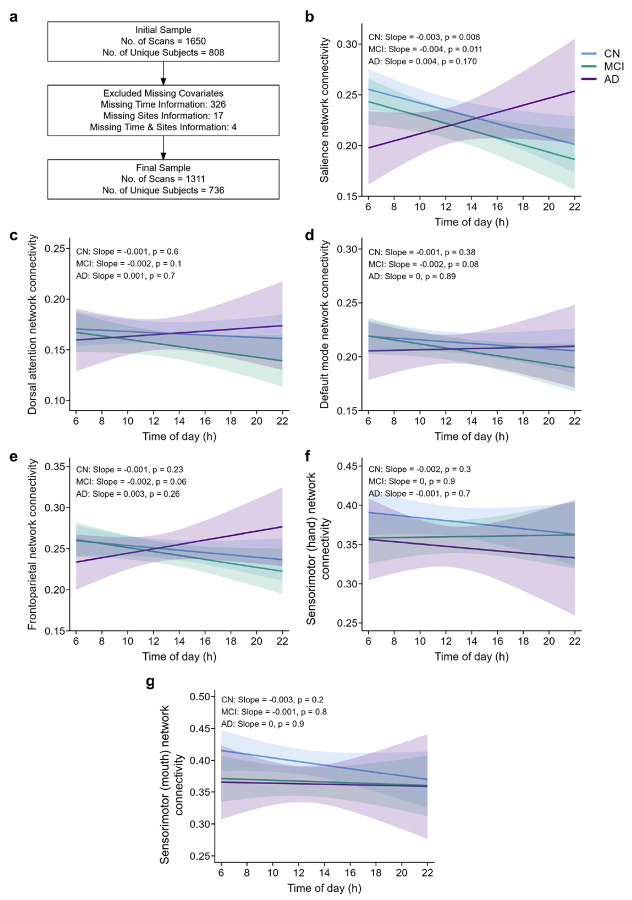
Time of day impacts salience network connectivity differentially based on cognitive status. **(a)** Flow chart of the included subjects in the study with resting state functional magnetic resonance imaging (rs-fMRI) data (n = 397, cognitively normal, CN; n = 248, mild cognitive impairment, MCI; n = 91, Alzheimer’s disease, AD). **(b-g)** Functional connectivity of the **(b)** Salience network**, (c)** Dorsal attention network, **(d)** Default mode network, **(e)** Frontoparietal network**, (f)** Sensorimotor (hand) network, **(g)** Sensorimotor (mouth) network **v**in CN, MCI, and AD subjects across the time of day. Shaded portions denote the 95% confidence interval. There is an interaction effect between cognitive status (AD diagnosis) with Salience network connectivity over time of day (AD diagnosis x Time, p = 0.015). Assessed using a mixed linear model controlling for several covariates (see Methods).

We next hypothesized that time of day differentially impacts functional connectivity in AD subjects and analyzed a large-scale rs-fMRI dataset consisting of 1311 scans from the Alzheimer’s Disease Neuroimaging Initiative (ADNI) (Fig. 6a). Several functional networks are affected in AD, including the Salience (SN), Default mode (DMN), and Frontoparietal (FPN) networks^64-67^. Therefore, we assessed time-of-day effects on resting-state functional connectivity (rs-FC) in the SN, DMN, FPN, as well as the dorsal attention (DAN) and sensorimotor networks. Although scan times were not prescribed, we leveraged the natural distribution of scan times for this analysis (Supplementary Fig. 7a). Participants were stratified by cognitive status into cognitively normal (CN), mild cognitive impairment (MCI), and AD groups (see Extended Data Table 1 for subject demographics). For each network, we tested associations between scan time, cognitive status, and rs-FC using a linear mixed-effects model (LMEM) (See Methods for covariates).

Overall, rs-FC did not vary with time of day (Supplementary Fig. 6a-f). However, the SN showed a significant time-of-day interaction with cognitive status (Fig. 6b-g; Extended Data Table 2)—whereas CN and MCI showed decreased connectivity later in the day, AD subjects showed the opposite. While an exploratory sex-stratified analysis showed this effect was stronger in male subjects (Supplementary Fig. 7b,c), a three-way LMEM model revealed no significant sex interaction (Supplementary Table 3). We then investigated the overlap between SN nodes and regions identified in our mouse activity-mapping studies. While direct human–mouse neuroanatomical mapping is limited by differences in cortical folding and evolutionary divergence^68-70^, we used established functional and circuit-based studies^71-74^ to identify node correlates for the SN in mice (Extended Data Table 3). Several key mouse regions identified in our mouse analyses (∣BR∣ > 2 from PLSC or Cluster 1 regions) corresponded to the SN correlates, including the ACA and PL. Using a cross-species approach, our data converge to identify nodes for future targeted investigation and potential therapeutic intervention.

## Discussion

Here, we developed a model of sundowning with face validity by characterizing sleep disruption and performing widescale behavioral phenotyping at Sundown and Sunrise using traditional and computational approaches for characterization. We report, for the first time, a novel signature of agitated, repetitive head-turning and pressured exploration selectively at Sundown in AD mice. Although internal affect cannot be confirmed in mice, we propose this behavior could model nighttime wandering seen in many sundowning patients. Our analyses further support unsupervised pose-tracking as an effective strategy for categorizing and predicting sundowning behaviors, which has potential for future clinical application. We expand on behavioral findings by mapping altered circadian and brain-wide activity patterns in AD mice and by discovering a time-of-day-dependent rs-FC alteration in the SN of AD subjects.

Our activity recording and EEG findings demonstrate disrupted sleep and circadian processes in AD mice, with steeper decline of sleep-wake amplitude with age and reductions in NREM sleep time. Consistent with other studies^8,75-77^, delta power was diminished in aged AD mice and, as in humans, NREM deficits correlated with hippocampal and cortical Aβ pathology^10,11,78^. Notable, sleep-wake and sleep architecture disruptions appeared as early as 2 months in AD mice, aligning with findings of others using similar AD models^30,77,79^. Despite the lack of Aβ aggregates at this stage, subtle pathology may underlie these changes; for example, Yang et al. reported elevated p-Tau Thr23 in the SCN of 2-month-old mice^77^. These findings reinforce that sleep alterations may serve as early biomarkers in AD^10,11,78,80^ and future longitudinal studies may reveal if these early changes are predictive of sundowning-like behavior. A limitation is that recordings occurred under light–paced conditions, and we were unable to distinguish the contributions of disrupted circadian versus sleep circuitry, although both processes are likely disrupted in dementia^5,6^. Future work should disentangle these independent contributions using constant darkness conditions.

In the SCN, we found that AD mice failed to show a decrease in activation proportion of AVP^+^ cells between Sunrise and Sundown, indicating disrupted diurnal oscillation in a cell population critical for circadian pacing. The ventral SCN, which receives light through the retinohypothalamic tract^81^, mediates photic entrainment, whereas the dorsal, AVP-expressing SCN maintains internal rhythmicity and signals to hypothalamic targets^82^. These results suggest AD pathology disrupts intrinsic SCN pacing rather than light entrainment. Consistent with this interpretation, a recent study showing accelerated, not impaired photic re-entrainment and hypersensitivity to light in the 3xTg AD model^83^.

We next examined brain-wide activation patterns covarying with EPM behavior at Sundown. Regions most contributory to the behavior–brain relationship were linked to decision-making, attention, goal direction, sensory and motor processing, contextual memory, emotional regulation, and circadian control. We highlight, the ACA and PL, key SN node homologs, as regions showing strong contributions to behavior at Sundown, and identify sensorimotor hyper correlation as a possible signature of heightened sensitivity to sensory stimuli at Sundown.

Our human fMRI findings show increased SN connectivity with time of day in AD subjects. The SN filters internal and external stimuli to guide attention^84,85^, suggesting there is difficulty orienting to relevant environmental stimuli towards the end of the day in AD subjects. Our results complement prior studies correlating SN connectivity with psychomotor agitation in AD^64^ and showing SN connectivity is modulated by Aβ and sleep disturbances^86^. We caution that, while our mouse and human results are complementary, they differ in temporal resolution: fMRI results reflect gradual time-of-day changes, whereas mouse IEG mapping capture an acutely agitated state. Thus, additional regions beyond the SN may contribute to sundowning but remain undetectable without rs-FC measurements during bouts of sundowning. For example, heightened sensorimotor and striatal engagement, likely reflects the motor differences between Ctrl and AD mice and suggests differential sensitivity to environmental stimuli. In contrast, human fMRI requires stillness, potentially selecting out hyperactive individuals who might display similar sensorimotor effects. Overall, the culmination of our mouse and human data suggest sundowning represents an agitated state driven by impaired gating/processing of sensory streams and dysregulated attention networks.

Contextualizing our findings with prior work, few studies have performed behavioral batteries at different times of day in aging or AD models. One study reports decreased open arms time in the EPM with age in male APP mice^87^, differing from our results, potentially due to background strain. Previous work in male mice showed circadian control of aggression through a polysynaptic circuit between the SCN, SBPV, and ventrolateral portion of the ventromedial hypothalamus^57^. A more recent study showed TAPP mice, expressing Aβ and tau pathology, exhibit phase-delays and increased aggression; this was linked to tau pathology in lateral parabrachial dynorphin cells, which developed earlier in females^22^. We expand on these studies by 1) focusing on a wider-scale screening across ages and sex groups; 2) using unsupervised behavioral characterization; 3) performing brain-wide mapping activity at Sundown, and 4) finding a translationally analogous connectivity signature in humans.

Regarding sex differences, we conclude that the Sundown-Sunrise phenotype is similar in male and female AD mice, excepting earlier symptom presentation in females, potentially due to earlier pathology accumulation^88,89^. Given this similarity, we focused on 12-month-old females, the youngest group with behavioral disruption. Clinical findings on sex effects in sundowning remain inconsistent^17,19,90^, and it remains unknown if sex differences exist in the neurobiology of sundowning. Additionally, emerging data show different expression patterns amongst IEG markers, such as Arc, c-Fos, and Npas4^91-93^. Whether mapping c-Fos expression at Sundown and Sunrise compared to Arc would yield similar or differential insights is an open question. Overall, the convergence of sleep, circadian, behavioral, and network-level changes across mice and humans implicate sundowning as a presentation of dysregulated internal state-regulation, offering a mechanistic framework informing development of targeted interventions in the future.

## Methods

### Ethics

Experiments were approved by the Institutional Animal Care and Use Committees (IACUC) at the New York State Psychiatric Institute (NYSPI) dba Research Foundation for Mental Hygiene, Inc. (RFMH) under protocol no. 1640 or at Columbia University Irving Medical Center (CUIMC) under protocol no AC-AABL9550. All key resources are listed in **Supplementary Table 4.**

### Mice

Mice were bred in-house and kept on a 129S6/SvEv background after backcrossing to this strain for more than 10 generations. Double transgenic APPswe/PSEN1δE9 mice^23^ (AD) carrying the Swedish mutation in APP and the ΔE9 mutation in human presenilin 1 (PS1) were used. For activity tagging experiments, a triple transgenic line was generated as previously described^94^, where female AD^23^ (+) x ArcCreER^T2 25^(+) x R26R-STOP-floxed-enhanced yellow fluorescent protein (eYFP)^(f/f) 95^ mice were bred with male R26R-STOP-floxed-eYFP^(f/f) 95^ mice. Mice were kept on a 12 h ON/OFF light-dark cycle (lights ON, 06:00-18:00 h) with *ad libitum* access to food and water.

### Genotyping

The APP and PS1 genotyping were performed as previously described^94^ and as described on the Jackson Laboratory website (MMRRC Strain #034829-JAX). Genotyping of Cre and eYFP was performed as previously described^25^.

### Sleep-wake activity using Piezo-sleep boxes

Sleep-wake activity was measured using a non-invasive automated piezoelectric recording of movements and breath rate (PiezoSleep, Signal Solutions, LLC, Lexington, KY) situated in a light-controlled room on a standard 12 h light cycle (lights ON, 06:00-18:00 h) with continuous data collection. The light was ~300 lux during the light phase and ~0 lux during the dark phase in the room. Mice were individually housed in chambers equipped with sawdust bedding, with *ad libitum* access to food and water, and an initial 24 h acclimation period was followed by data recording for 120 h (5 consecutive 24 h cycles). Following recordings, mice were placed back into their home cages. Each cage rested on a polyvinylidene difluoride (PVDF) square sensor (17.8 × 17.8 cm, 110 μm thick) protected by a thin plastic tray (50.8 μm)^96^. A rubber pad between each sensor and the base prevented crosstalk between the cages. The sensors were connected to an amplifier, and pressure signals and breath rates were classified as movements related to activity and inactivity or sleep and wake. Sleep percentage and sleep bout length data were exported using SleepStats2p10 (Signal Solutions, LLC, Lexington, KY) software. Activity data, scaled to arbitrary units between 0.0-0.3, were extracted in 10 min bins using ActivityStatistics (Signal Solutions, LLC, Lexington, KY) software. Cosinor analysis to estimate amplitude and acrophase across 5 full days of recordings was performed using ClockLab software (v6.1, Actimetrics, Wilmette, IL).

### Surgical procedures

Mice were anesthetized with a mixture of ketamine and xylazine (100 mg/kg and 10 mg/kg, intraperitoneally, i.p.), then placed on a stereotaxic frame with a closed-loop heating system to maintain body temperature. A 1 cm skin incision was made on the dorsal aspect of the head, and three surgical stainless-steel screws were implanted as electrodes. The first screw was positioned 1.5 mm right to the midline and 1.5 mm anterior to the bregma; the second screw was placed 2 mm from midline and 1.5 mm posterior to the bregma; the third was inserted on top of the left cerebellum. Two stainless steel electromyography (EMG) electrodes were inserted into the neck musculature. Electroencephalography (EEG) screws and EMG electrodes were connected to a PCB board which was soldered with a 5-position pin connector. All the implants were secured onto the skull with dental cement (Lang Dental Manufacturing). Carprofen (5 mg/kg, subcutaneous, s.c.) was administered after surgery and daily for 3 days post-surgery. After surgery, the animals were returned to their home cages for recovery for at least 2 weeks before experimentation.

### Electroencephalography (EEG) recordings

Recordings were performed for 24 h (lights ON, 07:00-19:00 h) in a behavioral chamber inside a sound-attenuating cubicle (Med Associates Inc, Fairfax, VT). Animals were habituated in the chamber for at least 4 h before recording. EEG and EMG signals were recorded, bandpass filtered at 0.5–500 Hz and digitized at 1017 Hz with 32-channel amplifiers (TDT, PZ5, and RZ5D or Neuralynx Digital Lynx 4 S). For sleep analysis, spectral analysis was carried out using a fast Fourier transform (FFT) over a 5 s sliding window, sequentially shifted by 2 s increments (bins). Brain states were semi-automatically classified into wake, NREM sleep, and REM sleep states using a custom-written MATLAB program using the following scoring criteria: wake: desynchronized EEG and high EMG activity; NREM: synchronized EEG with high-amplitude, delta frequency (0.1-4 Hz) activity and low EMG activity; and REM: high power at theta frequencies (4–10 Hz) and low EMG activity. Semi-auto classification was validated manually by trained experimenters. Power for a given frequency was first normalized against average total power across the range 0.1–50 Hz and then log transformed using a base of 2. Power comparisons across the delta (0.1-4 Hz), theta (4-10 Hz), alpha (10-13 Hz), beta (13.1-30 Hz), and gamma (30.1-50 Hz) ranges were calculated by taking the area under the curve (AUC) of normalized power per range.

### Drugs and compounds

#### Methoxy-X04

Aβ-plaque pathology was visualized using Methoxy-X04 (Tocris Bioscience #4920, Minneapolis, MN) as previously described^97^. A 1 mg/mL solution was prepared by combining 45% propylene glycol, 45% 1X phosphate buffered saline (1X PBS), 10% dimethylsulfoxide (DMSO), and 10 mg of Methoxy-X04. Mice were injected intraperitoneally (i.p.) with 0.2 mL of Methoxy-X04 solution 24 h before euthanasia.

#### 4-Hydroxytamoxifen (4-OHT)

Recombination was induced with 4-OHT (Sigma, St Louis, MO). 4-OHT was dissolved by sonication in 10% EtOH/90% corn oil at a concentration of 10 mg/ml. One injection of 200 μl (2 mg) was intraperitoneally (i.p.) administered to each mouse.

#### Brexpriprazole

A 0.01 mg/ml solution of brexpiprazole (BOC Sciences, Shirley, NY) was prepared using sonication in a 10% 2-hydroxypropyl-beta-cyclodextrin solution. A 0.1M solution of HCl was used to lower the pH of solution to aid dissolution. Drops of 0.1M NaOH were then added to bring the solution up to a pH of 4.

### Behavioral assays

#### Open Field (OF)

For behavioral assays, mice were tested at sundown and sunrise: Sundown was operationally defined as the last two hours of the active period, or zeitgeber hours 22-0 (ZT22-0), whereas Sunrise was defined as the first two hours of the active period or zeitgeber hours 12-14 (ZT12-14). Motor and exploratory activity was quantified in 50 cm × 50 cm open field boxes constructed from PVC foam boards. Mice were individually placed in the center of the OF box and allowed to explore for 10 min. Videos were scored by ANY-maze behavioral tracking software (v.7.47, Stoelting, Wood Dale, IL) to measure the distance traveled and time spent in the center and periphery zones. The center and periphery zones were defined by creating a 5 x 5 evenly spaced grid, with each grid square comprising a 10 cm x 10 cm area. The centermost 3 x 3 squares were defined as the center and the remaining outermost square were defined as the perimeter. The number of rearing bouts was manually scored in a blind manner.

#### Elevated Plus Maze (EPM)

Mice were tested in a custom-built acrylic plus-shaped apparatus consisting of four arms, two open and two enclosed by walls. The apparatus was raised by a central platform to a height of 46 cm from the floor. Each arm was 26.5 cm long and 8 cm wide. Mice were individually placed in the center of the maze facing an open arm and were allowed to explore the maze for 5 min. Videos were scored by ANY-maze behavioral tracking software (v.7.47, Stoelting, Wood Dale, IL) to measure the amount of time and number of entries into the closed arms, open arms, and center of the EPM. Head-dipping behavior was automatically quantified by drawing rectangular zones of interest bordering each edge of the open arms. The number and duration of head entries into each of the edge-bordering zones were used as metric of number of head dips and duration. Scoring was qualitatively assessed for accuracy during tracking.

A subset of mice exhibited complete absence of exploration of the apparatus similar to freezing behavior, which has been previously described^31^. We considered these mice outliers. We screened out mice that did not show any investigative behavior between any zones of the apparatus upon first exposure. A mouse was only defined as an absent explorer if they met two simultaneous criteria: 1) they spent more than 180 consecutive seconds (3 min) in the same position and 2) over 95% of the total test time (>285 s) was spent in the same zone. In total, 12 trials out of 460 were removed (2.6% of runs), and there was no statistical difference in the in proportion of “absent explorers” to “explorers” in either the Ctrl or AD groups across all age groups.

For behavioral comparisons of first-time apparatus exposure to the EPM in 12-month-old female mice, we pooled all first exposure trials from Ctrl and AD mice, including those crossed to the ArcCreER^T2^ x eYFP genotype that were used for IEG activity tagging. Since 4-OHT is injected following behavioral testing, testing conditions are the same as that of non-activity tagged mice. Additionally, the ArcCreER^T2^ x eYFP genotype does not interfere with behavioral phenotype of AD mice^94,97^.

#### Immediate Early Gene (IEG) activity tagging

Immediately following behavioral testing (day 1), mice were injected with 200 μl (2 mg) of 4-OHT (i.p.) and dark-housed continuously for 3 days (days 2-4). Mice were taken out of the dark on the morning of day 4, cages were changed, and they were returned to the normal colony room to acclimate prior to behavioral testing. All precautions to prevent disturbances to the ArcCreER^T2^ x eYFP mice during dark housing were taken to reduce off-target labeling. Following the second behavioral trial, mice were euthanized 60 min after the start of the test to allow for staining of c-Fos protein expression.

#### Statistical analyses

Behavioral data were analyzed with Prism v.9.0 or v.10.0 using an alpha value of 0.05. Generally, main effects and interaction effects of Age and Genotype or Period (Sundown or Sunrise) were assessed with a two-way analysis of variance (ANOVA) and followed up with Tukey or Sidak’s multiple comparisons testing. Statistical comparison of syllable frequencies was performed in R (v 4.5.0) using a three-way mixed ANOVA, with Genotype and Period as independent factors, and Syllable ID as a within-subjects factor. See **Legends** for exact details of statistical and post-hoc tests used. Because our multifactorial ANOVAs yielded multiple main and interaction effects, in the Results and **Figures** we report only significant effects and post-hoc findings that are critical for data interpretation. However, all statistical values, post-hoc tests, and associated p-values for Fig. 1-5, Extended data figures 1-7, and Supplementary figures 1-2 are listed in **Supplementary Table 1.**

#### Pose-tracking and unsupervised behavioral analysis

##### Video recordings and preprocessing:

Videos were recording in a top-down orientation using a Logitech C920S Pro HD webcam at a resolution of 640p x 480p at 30fps. EPM recordings were triggered per trial with ANY-maze software (v7.47, Stoelting, Wood Dale, IL), saved as .szv files, and then exported as .mp4 files using the highest quality compression setting. HandBrake software (v1.9.2) was used to standardize distances by cropping each video manually such that the field of view (FOV) fit the apparatus symmetrically and achieve an average distance-to-pixel ratio of 1cm = 6.5 p and a final video resolution of 480 p x 480 p. To make each frame reliably seekable prior to pose estimation, all videos were losslessly re-encoded at 30fps using the H.264 codec with FFmpeg software (v2024-02-22-git-76b2bb96b4).

##### Pose estimation:

SLEAP software (v1.3.0)^37^ was used to label and predict ten distinct body parts: nose, base of neck, left ear, right ear, left shoulder, right shoulder, center, left flank, right flank, tail base. A manually trained annotator labelled 446 frames distributed across all behavioral videos of performance in the EPM. Over 400 labelled instances were used to train a single instance pose estimation model using a UNET neural network architecture (hyperparameters: max stride = 16, filters = 16) for 200 epoch and validation was tested on 45 frames. The average body part distance (ground truth vs. prediction) was 5.5 pixels. Once labels were predicted on all frames, each video was manually inspected for qualitative accuracy of pose-tracking prior to export as an .H5 file for downstream analysis.

##### Unsupervised extraction of behavioral syllables using Keypoint-Moseq:

Keypoint-MoSeq (KPMS, v0.4.7)^38^ was used to identify behavioral syllables using 11 latent dimensions (>= 90% variance explained). A kappa hyperparameter of 5e10 was used for fitting the initial autoregressive hidden Markov model fitting (AR-HMM) for 50 iterations, whereas a kappa value of 1e3 was used to fit the full KPMS model for 400 iterations. Grid videos, trajectory plots, and summary statistics on kinematic information and syllable frequencies were exported using KPMS functions.

#### Linear classification of behavioral metrics

Classification of period and genotype subgroups based on behavioral metrics was performed using multinomial logistic regression in R (v 4.5.0) with the tidymodels package (v 1.30). The predictors used were either standard behavior metrics, including total distance traveled, number of head dips, total open time, total center time, and total closed arm time or KPMS syllable frequencies. Eight-fold cross validation with 200 repeats were performed. Folds were stratified by class to ensure an even representation of class labels in the training and held out sets. Predictions made on KPMS shuffled data were generated by randomly shuffling class labels 50 times and performing 8-fold cross validation with 4 repeats per shuffle (50 x 4 = 200 randomized repeats). Confusion matrices were then generated based on all classified labels across 200 repeats. Matrices were indexed such that rows represented the true class of a prediction, whereas columns represented classified label of the prediction. Matrix elements were then divided per row by the sum of each row (i.e., total of each true class), to generation prediction probabilities (predicted class as a proportion of the true class). ROC curves were created by plotting the false-positive rate (1-specificity) against the true-positive rate (sensitivity), using the roc_curve() function.

Precision, recall, and F_1_ scores summarize overall model performance and were computed per class for each predictor type based on the number of true positive (TP), false positives (FP), and false negatives (FN) classified. Precision = TP/(TP + FP), whereas recall = TP/ (TP + FN). F_1_ scores were calculated as the harmonic mean of precision and recall scores: F_1_ = 2 x (precision x recall) / (precision + recall). Confidence intervals (CI) for F_1_ scores were calculated per class by using the mean and standard error of all F_1_ scores and applying the student’s *t*-distribution to compute the CI bounds (based on the n - 1 degrees of freedom and the specified alpha value of 0.01).

### Brain processing

Mice were deeply anesthetized and transcardially perfused as previously described^98^. For SCN staining experiments, mice were perfused at either Sundown (ZT23) or Sunrise (ZT13) under dim red light (<3 lux) to avoid effects of additional light exposure during dark period on cell activation in the SCN. Brains were extracted and postfixed overnight in 4% paraformaldehyde (PFA) at 4°C, and cryoprotected in 30% sucrose / 1X phosphate buffered saline (PBS) solution at 4°C. Serial coronal sections (60 μm for brain-wide activity mapping; 50 μm for SCN staining) were cut using a vibratome (Leica VT1000S, Leica Biosystems, Nussloch, Germany) and stored in a 0.1 M PBS / 0.1% NaN_3_ solution at 4 °C.

### Immunohistochemistry

For activity mapping experiments, cells expressing eYFP and c-Fos were stained using a modified iDISCO-based protocol^99^. Briefly, sections were washed in 1X PBS 3 times for 10 min each, then dehydrated in 50% MeOH for 2.5 h. After, sections were washed in 0.2% Tween-20 in PBS (0.2% PBST) 3 times for 10 min each, and then blocked in a solution of 0.2% PBST, 10% DMSO, and 6% normal donkey serum (NDS) for 2 h. Sections were washed in 1X PBS, 0.2% Tween-20, and 10 μg heparin (PTwH), and then incubated in a primary antibody solution of rat polyclonal anti-c-Fos (1:5000, SySy, Goettingen, Germany) and chicken polyclonal anti-GFP (10 mg/ml, 1:2000, Abcam, Cambridge, MA) in PTwH / 5% DMSO / 3% NDS for 48 h at 4°C. Sections were then washed 3 times in PTwH for 10 min each, before being incubated in secondary antibody solution consisting of Alexa 647 conjugated donkey anti-rat IgG (1:500, Abcam, Cambridge, MA) and Cy2 conjugated donkey anti-chicken IgG (1:250, Jackson ImmunoResearch, West Grove, PA) in 3% NDS / PTwH overnight. The next day, sections were washed in 3 increments of 10 min each in PTwH, then washed in 3 increments of 10 min each in 1X PBS. Sections were then mounted on slides and allowed to dry for approximately 20 min before adding mounting medium Fluoromount G (Electron Microscopy Sciences, Hatfield, PA) and a coverslip.

For SCN subpopulation staining, sections were washed 3 times for 15 min in 1X PBS 0.1% Triton X-100 (0.1% PBSTx), followed by blocking in a solution of 0.3% PBSTx / 5% NDS for 1 h. Sections were incubated in primary solution of rat polyclonal anti-c-Fos (1:5000, SySy, Goettingen, Germany), rabbit polyclonal anti-VIP (1:10000, Immunostar, Hudson, WI), and mouse monoclonal anti-AVP (1:200, Santa Cruz Biotechnology, Dalla, TX) in 0.3% PBSTx / 1% NDS at 4 °C for 72 h. Sections were washed 3 times in 0.1% PBSTx for 10 min, before incubation in a secondary antibody solution of Alexa 647 conjugated donkey anti-rat IgG (1:500, Abcam, Cambridge, MA), Alexa 488 donkey anti-rabbit IgG (Invitrogen, Waltham, MA), Cy3 whole donkey anti-mouse IgG (1:500, Jackson ImmunoResearch Laboratories, West Grove, PA) in 0.3% PBSTx for 2 h. Afterwards, sections were washed twice for 15 min each in 1 X PBS, followed by a 15 min wash in Hoechst 33342 (1:10,000, Thermo Fisher Scientific, Waltham, MA) in 1X PBS. Sections were then mounted on slides and allowed to dry for approximately 20 min before adding mounting medium Fluoromount G (Electron Microscopy Sciences, Hatfield, PA) and a coverslip.

### Confocal microscopy

Coronal brain sections were imaged using a confocal scanning microscope (Leica TCS SP8, Leica Microsystems Inc., Wetzlar, Germany) with 2 simultaneous PMT detectors. Sections were imaged with a dry Leica 20x objective (NA 0.60, working distance 0.5 mm), with a pixel size of 1.08 x 1.08 μm^2^, a z step of 3 μm, and z-stack of 9 μm. Fields of view were stitched to form tiled images using an automated stage and the LAS X statistical blending algorithm with multi-frame search.

For brain activity mapping experiments, fluorescence from Cy2 was excited at 488 nm and detected at 500-550 nm, and Alexa Fluor 647 was excited at 638 nm and detected at 650-700 nm. Laser power was held constant between all sections. Raw images were saved as .lif files storing two channels: Cy2 (eYFP, channel 1) and Alexa Fluor 647 (c-Fos, channel 2).

For SCN subpopulation imaging, sections were imaged with the same resolution and a z step of 3 μm, and z-stack thickness of 27 μm. Sequential scanning was used to first image fluorescence from Hoechst 33342 (excited using a 405 nm UV laser, and detected at 420-480 nm), and Cy3 (excited at 552 nm and detected at 550-590 nm). For the second sequence, Alexa 488 was excited at 488 nm and detected at 500-540 nm, and Alexa 647 was excited at 638 nm and detected at 650-690 nm.

### Plaque burden quantification

A custom FIJI/ImageJ macro was used to automatically detect proportion of plaque positive pixels within a manually drawn region of interest (ROI). An average of 4 coronal sections were analyzed per mouse. Using the ROI Manager, an ROI was drawn around the left and right hippocampus per section. For the cortex, an ROI was drawn around all cortical regions contained in each section, without distinguishing subregions. Afterwards, background noise in each image was reduced using a median filter (5-pixel radius), followed by background subtraction using a rolling ball radius of 50 pixels. An absolute brightness threshold of 40 (out of a range 255) was applied, and the area of positively thresholded pixels and each ROI was calculated.

### SCN subpopulation cell quantification

Centermost SCN section were quantified to minimize variance from activity oscillation gradients along the rostro-caudal axis^100^. The boundaries of the SCN shell and core were manually drawn in ImageJ/FIJI (v 1.52p) using the ROI manager. The number of AVP^+^, VIP^+^, c-Fos^+^, and colabelled AVP^+^c-Fos^+^ or VIP^+^c-Fos^+^ cells in the SCN shell and core were manually counted by three annotators who qualitatively cross-checked counts for accuracy. c-Fos^+^ cells were counted using an automated cell counting approach previously cross-validated with manual counting^24^ (see Automated cell segmentation) and results were then qualitatively checked. Number of counts per channel were normalized by the area of either the core, shell, or total SCN area (core and shell combined). Colabelled AVP^+^c-Fos^+^ or VIP^+^c-Fos^+^ cells counts were divided by the total number of AVP^+^ or VIP^+^ counts, respectively, to calculate cell-type specific activation proportions.

### Automated cell segmentation

Extensive detail on the cell-segmentation algorithm steps and their validation have recently been published^24^. In brief, using a custom macro in ImageJ/FIJI, z-stack images of brain sections were converted from .lif to .tif files. Afterwards, since c-Fos localizes to the cell body whereas eYFP expression labels cell bodies as well as dendritic and axonal processes in the ArcCreER^T2^ x eYFP tagging system, a previously developed 3D segmentation approach was applied to each label due to these staining morphology differences^24,101^. The segmentation output was then qualitatively inspected per image, and over-segmentation errors due to structural autofluorescence artifacts (e.g., around ventricles and tissue edges) were manually deleted to maximize accuracy of mapping in the final dataset. Custom ImageJ/FIJI macros were written to apply preprocessing and segmentation algorithms in batch and all have been published^24^.

### Brain registration and mapping

Details of batch processing scripts to perform atlas registration and cell mapping have previously been published^24^. In brief, image z-stacks were automatically pre-processed using an ImageJ/FIJI macro to combine fluorescence intensities across both channels and z-planes into a flattened maximum project image. Images were manually assigned to a best-matching anterior-posterior atlas coordinate based on the reference atlas available at https://osf.io/cpt5w. Registration with flexible user-correction was then performed in R on these flattened images using SMARTTR^24^, which interfaces with the relevant registration functions in the WholeBrain^102^ and SMART^103^ packages. Cell counts of eYFP^+^ and c-Fos^+^ cells were warped to atlas space, and mapped cell counts were then normalized by region volume to yield cells per mm^3^.

### Behavioral partial least squares correlation

PLSC was performed in R independently for the sundown period (eYFP^+^) and sunrise period (c-Fos^+^) associated data using functions adapted from the packages data4PCCAR and PTCA4CATA (see **Code availability**). Conceptually, PLSC is similar principal component analysis (PCA), in that it addresses multicollinearity by collapsing correlated features into orthogonal latent components, each capturing distinct, non-redundant patterns of covariation. The statistical advantage of PLSC is its ability to detect brain–behavior associations by modeling distributed multivariate patterns across regions, thereby avoiding multiple independent regional comparisons^45,104,105^.

Brain data was first imputed to account for missing values. Regions with high amounts of missing data (>2 points per group per region) were removed from further analysis. The remaining missing data were assumed to be missing at random (MAR) and imputed using group-wise region median values. A correlation matrix was then generated as a product between behavioral features-–total open time, total center time, total closed time, total distance, and KPMS syllables 8, 10, 12, 13, 14, 16, and 18—and regional brain activations. This matrix was centered, scaled, then decomposed using singular value decomposition (SVD), with the tepPLS() function from the TExPosition package. This yielded pairs of latent variables, with corresponding brain and behavioral saliences representing the contribution (i.e., "weights") of each original feature to the latent dimensions.

To evaluate the significance and stability of the latent variables, we employed both permutation testing and bootstrapping^47^. For permutation testing, brain region labels were shuffled within subjects across 10,000 iterations to create a null distribution of singular values (i.e., the covariance strength captured by each latent dimension). Latent variables with singular values exceeding the 95th percentile of the permuted distribution were deemed significant. Bootstrapping was then performed by resampling the data with replacement for 10,000 iterations to generate bootstrapped saliences for each latent variable. Bootstrap ratios (BRs) were calculated by dividing the original salience values by their bootstrapped standard errors. These BRs are analogous to z-scores, where ∣BR∣ ≥ 2 approximates a 95% confidence interval, indicating reliable feature contributions to the respective latent variable.

### Regional correlation and network analysis

Cross correlations heatmaps and network analysis and visualizations were performed using the SMARTTR package^24^ in R. Pearson correlations between regions were calculated using the get_correlations() function and asymptotic p-values for each correlation were determined using a one-sample t-test. Significantly correlated regions were visualized in heatmaps using conservative alpha values of p < 0.005 (eYFP^+^) and p < 0.001 (c-Fos^+^). To better visualize differences in connectivity patterns amongst the strongest connections in the Ctrl and AD networks, we created a shared set of nodes between groups using the create_joined_networks() function. To generate sparsity, we proportionally filtered edge by p-value, retaining the most significant values, to achieve an edge density of 1.5%. To compare global network properties across a range of alpha thresholds, we use the create_networks() and summarise_networks() functions to calculate mean degree, mean efficiency, mean clustering, and mean betweenness across a range of thresholds from 0.001 to 1.

### Hierarchical clustering

Region cross-correlation matrices of eYFP^+^ expression in AD mice were turned into a dissimilarity matrix using the formula, distance = 1 - ∣r∣. Using this distance metric, both strongly positive and negative correlations were considered shorter distances, capturing coregulated activity, whereas weak or absent correlations had higher distances with a maximum value of 1. Hierarchical clustering was then performed using linkage based on average intercluster dissimilarity (unweighted pair group method with arithmetic mean, UPGMA). To determine the optimum dendrogram cut height and, therefore number of clusters (k), the sum of all within cluster distances was calculated for each k value across the maximum cluster range (1 to 137). Using the “elbow” heuristic, a k value of 7 was chosen due to diminishing reduction of total within cluster dissimilarity beyond this threshold.

### Human ADNI analysis

#### Participants

Data used in the preparation of this article were obtained from the Alzheimer’s Disease Neuroimaging Initiative (ADNI) database (www.adni.loni.usc.edu). The ADNI was launched in 2003 as a public-private partnership, led by Principal Investigator Michael W. Weiner, MD. The primary goal of ADNI has been to test whether serial magnetic resonance imaging (MRI), positron emission tomography (PET), other biological markers, and clinical and neuropsychological assessment can be combined to measure the progression of mild cognitive impairment (MCI) and early Alzheimer’s disease (AD). A detailed description of the ADNI cohort has been previously published^106^.

Final rs-fMRI data with covariates were acquired from 1311 scans from 736 unique participants, including 397 cognitively normal subjects (CN, 53.9%%), 248 subjects with mild cognitive impairment (MCI, 33.7%), and 91 subjects diagnosed with AD (12.4%). Qualifying MCI subjects had the following inclusion criteria: 1) memory complaints without significant functional impairment; 2) scores between 24 and 30 on the mini-mental status examination (MMSE); 3) a global clinical dementia rating (CDR) score of 0.5; 4) a CDR memory score of 0.5 or greater; or 5) objective memory impairment on the Wechsler Memory Scale – Logical Memory II test. Qualifying CN subjects had: 1) MMSE scores between 24 and 30; 2) a global CDR of 0; and 3) did not meet criteria for MCI or AD. Inclusion and diagnostic criteria, as well as procedures and protocols, for the ADNI studies can be found at https://adni.loni.usc.edu/data-samples/adni-data/study-cohort-information/. For the current study, we included individuals considered CN, MCI (both early and late MCI), and AD.

#### Standard protocol approvals, registrations, and patient consents

All procedures were approved by the Institutional Review Boards of all participating institutions. Written informed consent was obtained from every research participant according to the Declaration of Helsinki and the Belmont Report. For more up-to-date information, see https://adni.loni.usc.edu/help-faqs/adni-documentation/.

#### rs-fMRI network analysis

Functional data was preprocessed using fMRIPrep^107^(v 20.2.3). Specifically, a reference volume and its skull-stripped version were generated using the custom methodology of fMRIPrep. Head-motion parameters with respect to the BOLD reference (transformation matrices, and six corresponding rotation and translation parameters) were estimated before any spatiotemporal filtering using mcflirt^108^ (FSL 5.0.9). BOLD runs were slice-time corrected using 3dTshift from AFNI 20160207^103^. The BOLD reference was then co-registered to the T1w reference using bbregister (FreeSurfer v7.4.1) which implements boundary-based registration^110^. Co-registration was configured with six degrees of freedom. The BOLD time-series were resampled into standard MNI152NLin2009cAsym space. Several confounding time-series were calculated based on the preprocessed BOLD: Frame-wise displacement (FWD) was calculated from the six motion parameters and root-mean-square difference (RMSD) of the BOLD percentage signal in the consecutive volumes. Contaminated volumes were then detected and classified as outliers by the criteria FWD > 0.5 mm or RMSD > 0.3% and replaced with new volumes generated by linear interpolation of adjacent volumes. The three global signals were extracted within the cerebrospinal fluid (CSF), the white matter masks. A bandpass filter with cut-off frequencies of 0.01 and 0.09 Hz was used. Finally, the covariates corresponding to head motion (6 realignment parameters), outliers, and the BOLD time series from the subject-specific white matter and CSF masks were used in the connectivity analysis as predictors of no interest and were removed from the BOLD functional time series using linear regression.

Within-network functional connectivity was assessed using the Power atlas^111^, which defines 264 cortical and subcortical coordinates corresponding to functionally homogeneous brain regions. For each coordinate, a 6-mm radius spherical region was created, and the mean time series was extracted by averaging across all voxels within each sphere. Pairwise Pearson correlation coefficients were computed between the time series of all spheres, and functional connectivity was quantified as the Fisher’s z-transformed correlation coefficients among node pairs belonging to the same functional network. We examined the salience (SN), default mode (DMN), frontoparietal (FPN), somatomotor-hand (SMN-hand), somatomotor-mouth (SMN-mouth), and dorsal attention (DAN) networks.

### Statistical analysis of rs-fMRI data

Demographic characteristics between the CN, MCI, and AD subjects were compared using the ANOVA (for continuous variables) and chi-square tests (for categorical variables). Descriptive statistics were reported as means with standard deviations for continuous variables and as frequencies with percentages for categorical variables. We tested the association between scan time, cognitive status, and resting state functional connectivity (rs-FC) using a linear mixed-effects model (LMEM). Each LMEM regression included cognitive status (CN, MCI, AD), scan time (Time), their interactions, and covariates (age, gender, sedative or hypnotics usage, presence or absence of hypertension, and motion artifact [FWD]) as fixed effects and random intercepts of site and subjects to account for site differences and repeated scans from participants. For any significant between cognitive status x time interactions, post hoc contrast analyses were conducted to estimate group-specific time effects. All analyses were performed using R software (v 4.5.2), and p-values < 0.05 were considered to indicate statistical significance.

## Extended Data

**Extended Data Table 1 ∣:** Subject demographics. Cognitively normal, CN; mild cognitive impairment, MCI; Alzheimer’s disease, AD; standard deviation, SD. Significant main and interaction effects are bolded.

**Extended Data Table 2 ∣:** Main and interaction effects of cognitive state and time of scan across networks. Mild cognitive impairment, MCI; Alzheimer’s disease, AD; Salience network, SN; Default mode network, DMN; Frontoparietal network, FPN; Sensorimotor (hand) network, SMN (hand); Sensorimotor (mouth) network, SMN (mouth); Dorsal attention network, (DAN). Significant main and interaction effects are bolded.

**Extended Data Table 3 ∣:** Salience Network nodes and homologs in mice. Automated anatomical labelling atlas 3, AAL3; Montreal Neurological Institute Coordinates, MNI; Human connectome project atlas, HCP atlas; bootstrap ratio, BR; partial least squares correlation, PLSC. Bolded Allen Mouse Brain Atlas regions are ones which reached both a ∣BR∣ > 2 during PLSC analysis or a region found in Cluster 1 following hierarchical clustering of correlated activity in AD mice at Sundown. FOV. Bottom, red, the plaque-positive pixels detected automatically using a custom FIJI/ImageJ macro (see Methods). (d) No correlations were found between hippocampal (d) or cortical (e) plaque burden (% area) and normalized delta power during NREM sleep. * p < 0.05, * *p < 0.01. Error bars represent ± SEM.

## Supplementary Material

Supplement 1 **Supplementary Fig. 1 ∣ Automated plaque analysis in aged AD mice. (a)** Representative coronal image of absent Aβ-plaques plaque expression in a 2-month-old AD mouse. **(b)** Aβ-plaques stained with Methoxy-X04 in a 12-month-old AD mouse. **(c)** Inset of the region of the field of view (FOV) highlighted with white dotted lines in **b.** Top, green, the structure of the hippocampus using autofluorescence. A line indicates the regional outline of the hippocampus. Middle, blue, Methoxy-X04 expression in the same FOV. Bottom, red, the plaque-positive pixels detected automatically using a custom FIJI/ImageJ macro (see Methods). **(d)** No correlations were found between hippocampal **(d)** or cortical **(e)** plaque burden (% area) and normalized delta power during NREM sleep. * p < 0.05, * *p < 0.01. Error bars represent ± SEM.
**Supplementary Fig. 2 ∣ Brexpiprazole decreases aberrant exploratory activity at sundown in AD mice. (a)** Experimental design. Female 12-month-old Ctrl or AD mice were injected with vehicle (Veh) or 0.1 mg/kg of brexpiprazole (Brex) 1 h prior to behavioral testing in the OF or EPM at sundown. **(b)** Brexpiprazole administration decreased the distance traveled in the OF when compared to vehicle administration in AD, but not Ctrl mice at sundown. **(c)** Brexpiprazole administration decreased the time spent in the open arms in the EPM when compared to vehicle administration in AD, but not Ctrl mice at sundown. * p < 0.05, **p < 0.01. Error bars represent ± SEM.
**Supplementary Fig. 3 ∣ Registration and mapping to a standard atlas space. (a)** Labelling of Sunrise-active Arc+ (eYFP, green) and Sundown-active c-Fos+ (red) cells. **(b)** Right, a magnified inset of the hippocampus corresponding to the white dotted box on the left. **(c)** Grayscale images of coronal sections with eYFP^+^ and c-Fos^+^ signals combined (Flattened raw). Corresponding coordinates of the best matching Allen Mouse Brain atlas plates are displayed on the left. **(d)** The user-corrected registration and atlas overlay over the image. **(e)** Representative images of imported automatic cell-counts (c-Fos^+^) onto the registration overlay. Anterior-posterior, AP.
**Supplementary Fig. 4 ∣ Differential expression of eYFP^+^ activity across all mapped regions.** Volume normalized (cells / mm^3^) counts of sundown-active eYFP^+^ cells between Ctrl (white bars) or AD (green bars) mice across all regions mapped. Subregion activity expression patterns are organized by parent anatomical divisions. olfactory areas, OLF; hippocampal formation, HPF; cortical subplate, CTXsp; cerebral nuclei, CNU; thalamus, TH; hypothalamus, HY; midbrain, MB; hindbrain, HB. (n = 6-8 mice per group). Error bars represent ± SEM.
**Supplementary Fig. 5 ∣ Differential expression of c-Fos^+^ activity across all mapped regions.** Volume normalized (cells / mm^3^) counts of sunrise-active c-Fos^+^ cells between Ctrl (white bars) or AD (green bars) mice across all regions mapped. Subregion activity expression patterns are organized by parent anatomical divisions. olfactory areas, OLF; hippocampal formation, HPF; cortical subplate, CTXsp; cerebral nuclei, CNU; thalamus, TH; hypothalamus, HY; midbrain, MB; hindbrain, HB. (n = 6-8 mice per group). Error bars represent ± SEM.
**Supplementary Fig. 6. ∣ Network connectivity over time of day does not change across most functional brain networks. (a-f)** Resting-state functional connectivity does not change over time of day across all subjects (pooled across cognitive states) for the **(a)** dorsal attention network, **(b)** default mode network, **(c)** frontoparietal network, **(d)** sensorimotor (hand) network, **(e)** sensorimotor (mouth) network, and the **(f)** salience network. Individual values per scan are plotted as grey dots. The red lines represent the least-squares regression line per network.
**Supplementary Fig. 7 ∣ Sex stratified analysis of salience network connectivity over time of day across cognitive conditions. (a)** A density plot of showing the distribution of scan times in female subjects (pink) and male subject (blue). **(b-c)** Functional connectivity of the salience network in **(b)** female and **(c)** male CN, MCI, and AD subjects across time of day. Shaded portions denote the 95% confidence interval (CI). There is an interaction effect between cognitive status (AD diagnosis) with salience network connectivity over time of day in male (AD diagnosis x Time, p = 0.034), but not female subjects (AD diagnosis x Time, p = 0.300). Assessed using a mixed linear model controlling for several covariates **(b, c,** see Methods).

Supplementary Table 1 ∣ Statistics tables for main figures 1-5, extended figures 1-7, and supplementary figures 1-2.

Supplementary Table 2 ∣ Table of abbreviations.

**Supplementary Table 3 ∣ Main and interaction effects of cognitive state, time of scan, and gender across networks.** Mild cognitive impairment, MCI; Alzheimer's disease, AD; Salience network, SN; Default mode network, DMN; Frontoparietal network, FPN; Sensorimotor (hand) network, SMN (hand); Sensorimotor (mouth) network, SMN (mouth); Dorsal attention network, (DAN). Significant main and interaction effects are bolded.

Supplementary Table 4 ∣ Key Resources.

**Supplementary Video 1 ∣ Behavioral performance of a 12-month-old female Ctrl mouse in the EPM at Sundown.** The video is sped up 6x.

**Supplementary Video 2 ∣ Behavioral performance of a 12-month-old female AD mouse in the EPM at Sundown.** The video is sped up 6x.

**Supplementary Video 3 ∣ Grid video showing representative instances of syllable 15.** Qualitatively, syllable 15 resembles instances of entry into the dark arms of the EPM. This serves as an example of a KPMS syllables capturing a structured behavior which does not differentiate between Ctrl Sunrise, AD Sunrise, Ctrl Sundown, and AD Sundown groups. White dots appear on the centroid position of each mouse during the syllable onset and offset for each instance.

**Supplementary Video 4 ∣ Grid video showing representative instances of syllable 10.** Qualitatively, syllable 10 resembles instances of head retraction. White dots appear on the centroid position of each mouse during the syllable onset and offset for each instance.

**Supplementary Video 5 ∣ Grid video showing representative instances of syllable 13.** Qualitatively, syllable 13 resembles instances of a right head turn. White dots appear on the centroid position of each mouse during the syllable onset and offset for each instance.

**Supplementary Video 6 ∣ Grid video showing representative instances of syllable 14.** Qualitatively, syllable 14 resembles instances of a left head turn. White dots appear on the centroid position of each mouse during the syllable onset and offset for each instance.

Supplementary Files

This is a list of supplementary files associated with this preprint. Click to download.

- 7TableS1.pdf
- 7TableS2.pdf
- 7TableS3.pdf
- 7TableS4.pdf
- 8VideoS1.mp4
- 8VideoS2.mp4
- 8VideoS3.mp4
- 8VideoS4.mp4
- 8VideoS5.mp4
- 8VideoS6.mp4
- 5ExtendedTable1.pdf
- 5ExtendedTable2.pdf
- 5ExtendedTable3.pdf

## Data Availability

All raw imaging and behavioral datasets generated during this study are available from the corresponding author upon reasonable request. All custom analysis scripts used in this paper are available online at https://github.com/mjin1812/sundowning.
