# Supplementary material for: Behavioral and brain-wide neural signatures of sundowning in Alzheimer’s disease
Source: Res Sq. 2026 Apr 20:rs.3.rs-9272255. Preprint. [Version 1] doi: 10.21203/rs.3.rs-9272255/v1 (PMC13131861; doi:10.21203/rs.3.rs-9272255/v1)
Supplement: Supplement 1 [file NIHPPrs9272255v1-supplement-1.pdf]

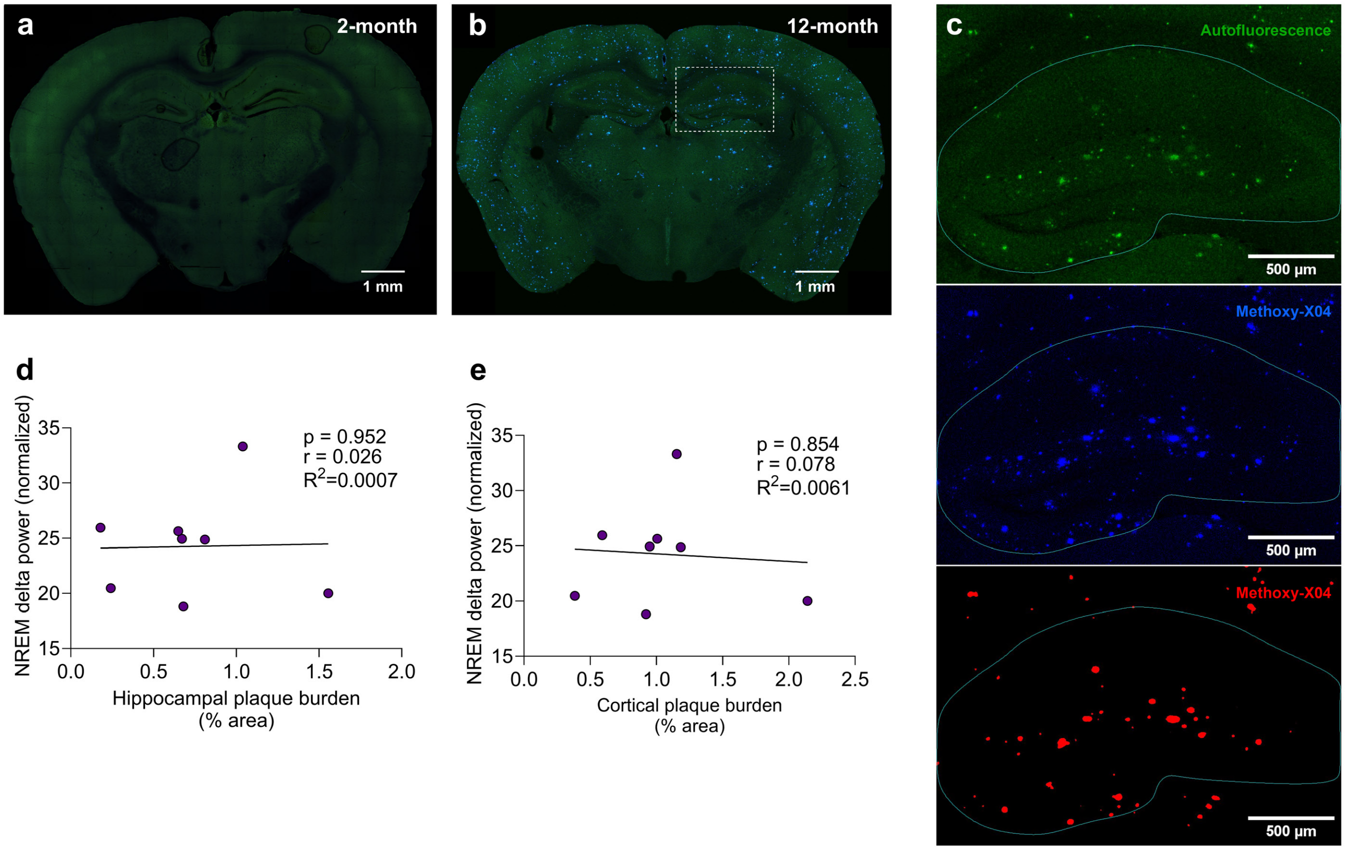

Supplementary Fig. 1 | Automated plaque analysis in aged AD mice. (a) Representative coronal image of absent Aβ-plaques plaque expression in a 2-month-old AD mouse. **(b)** Aβ-plaques stained with Methoxy-X04 in a 12-month-old AD mouse. **(c)** Inset of the region of the field of view (FOV) highlighted with white dotted lines in **b**. Top, green, the structure of the hippocampus using autofluorescence. A line indicates the regional outline of the hippocampus. Middle, blue, Methoxy-X04 expression in the same FOV. Bottom, red, the plaque-positive pixels detected automatically using a custom FIJI/ImageJ macro (see **Methods**). **(d)** No correlations were found between hippocampal **(d)** or cortical **(e)** plaque burden (% area) and normalized delta power during NREM sleep. * $p < 0.05$, * *$p < 0.01$. Error bars represent ± SEM.

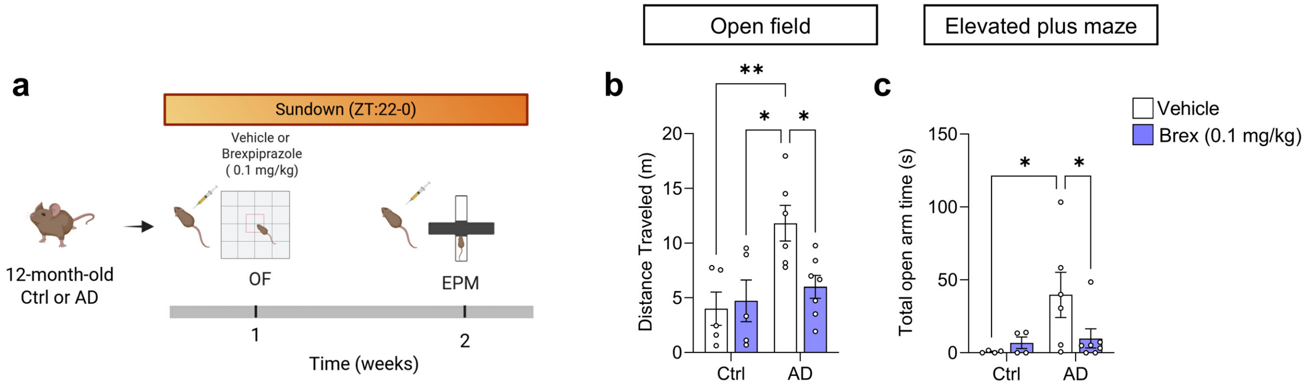

Supplementary Fig. 2 | Brexpiprazole decreases aberrant exploratory activity at sundown in AD mice. (a) Experimental design. Female 12-month-old Ctrl or AD mice were injected with vehicle (Veh) or 0.1 mg/kg of brexpiprazole (Brex) 1 h prior to behavioral testing in the OF or EPM at sundown. **(b)** Brexpiprazole administration decreased the distance traveled in the OF when compared to vehicle administration in AD, but not Ctrl mice at sundown. **(c)** Brexpiprazole administration decreased the time spent in the open arms in the EPM when compared to vehicle administration in AD, but not Ctrl mice at sundown. * $p < 0.05$, ** $p < 0.01$. Error bars represent ± SEM.

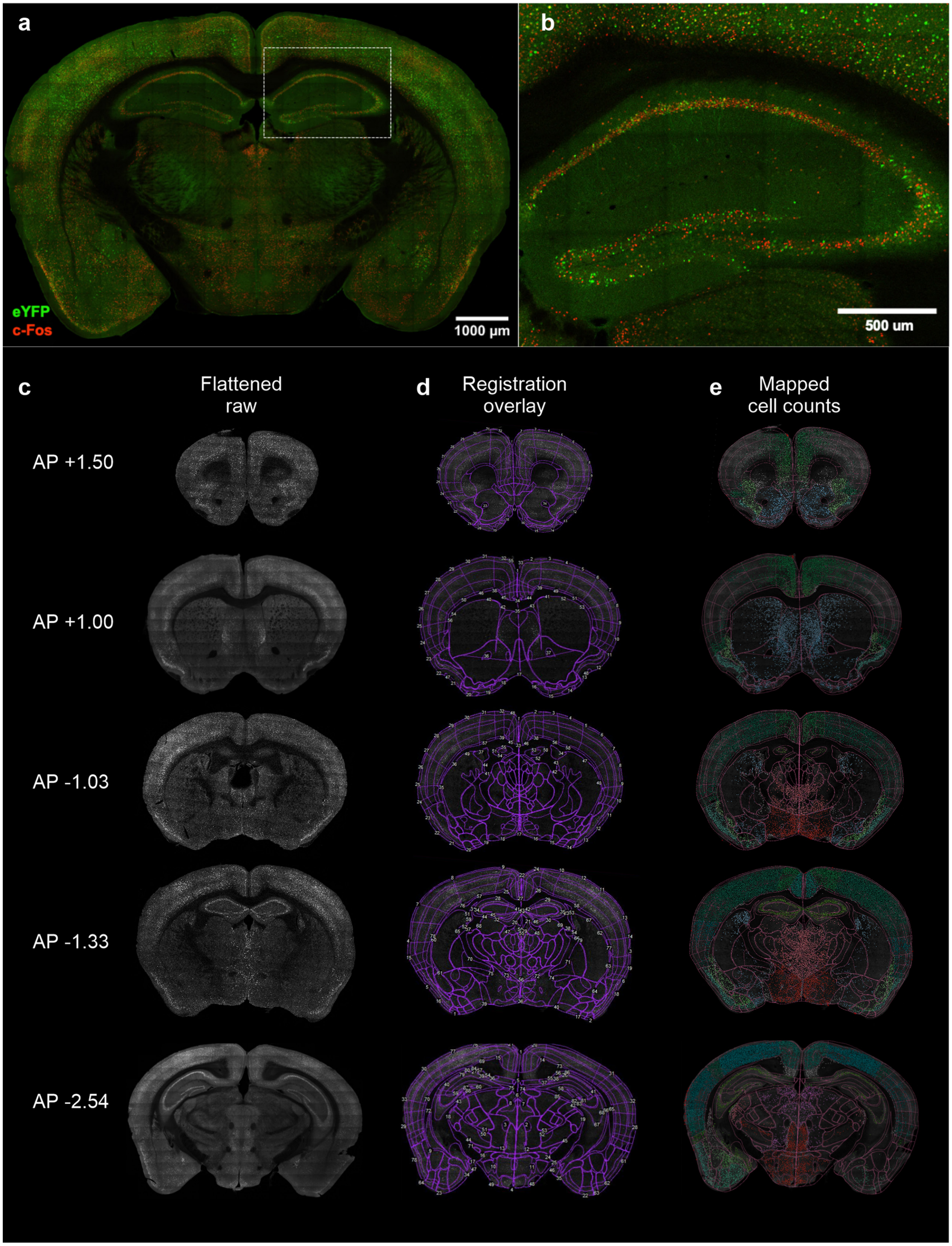

Supplementary Fig. 3 | Registration and mapping to a standard atlas space. (a) Labelling of Sunrise-active Arc+ (eYFP, green) and Sundown-active c-Fos+ (red) cells. **(b)** Right, a magnified inset of the hippocampus corresponding to the white dotted box on the left. **(c)** Grayscale images of coronal sections with eYFP^{+} and c-Fos^{+} signals combined (Flattened raw). Corresponding coordinates of the best matching Allen Mouse Brain atlas plates are displayed on the left. **(d)** The user-corrected registration and atlas overlay over the image. **(e)** Representative images of imported automatic cell-counts (c-Fos^{+}) onto the registration overlay. Anterior-posterior, AP.

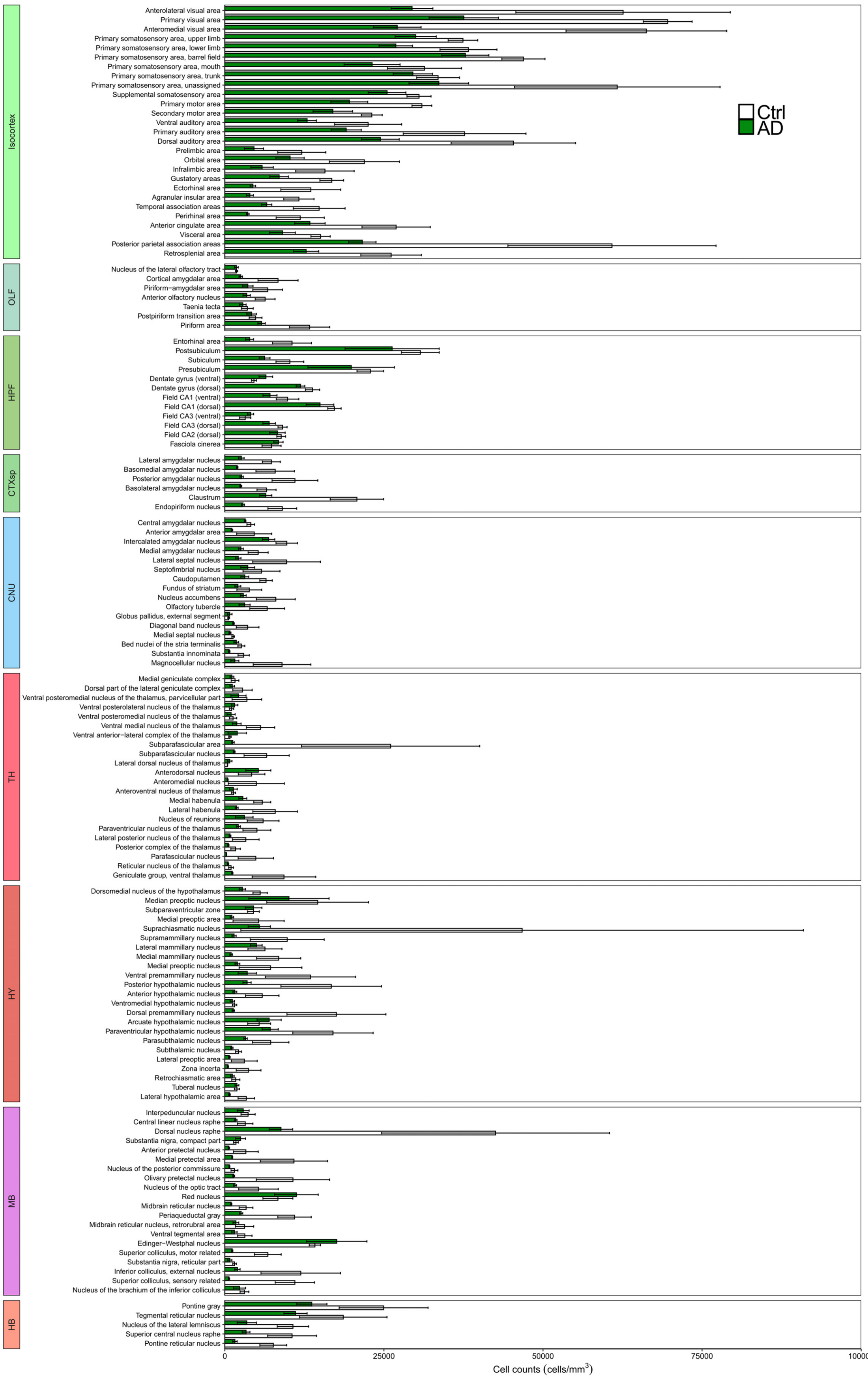

Supplementary Fig. 4 | Differential expression of eYFP$^+$ activity across all mapped regions. Volume normalized (cells / mm^3) counts of sundown-active eYFP$^+$ cells between Ctrl (white bars) or AD

(green bars) mice across all regions mapped. Subregion activity expression patterns are organized by parent anatomical divisions. olfactory areas, OLF; hippocampal formation, HPF; cortical subplate, CTXsp; cerebral nuclei, CNU; thalamus, TH; hypothalamus, HY; midbrain, MB; hindbrain, HB. (n = 6-8 mice per group). Error bars represent ± SEM.

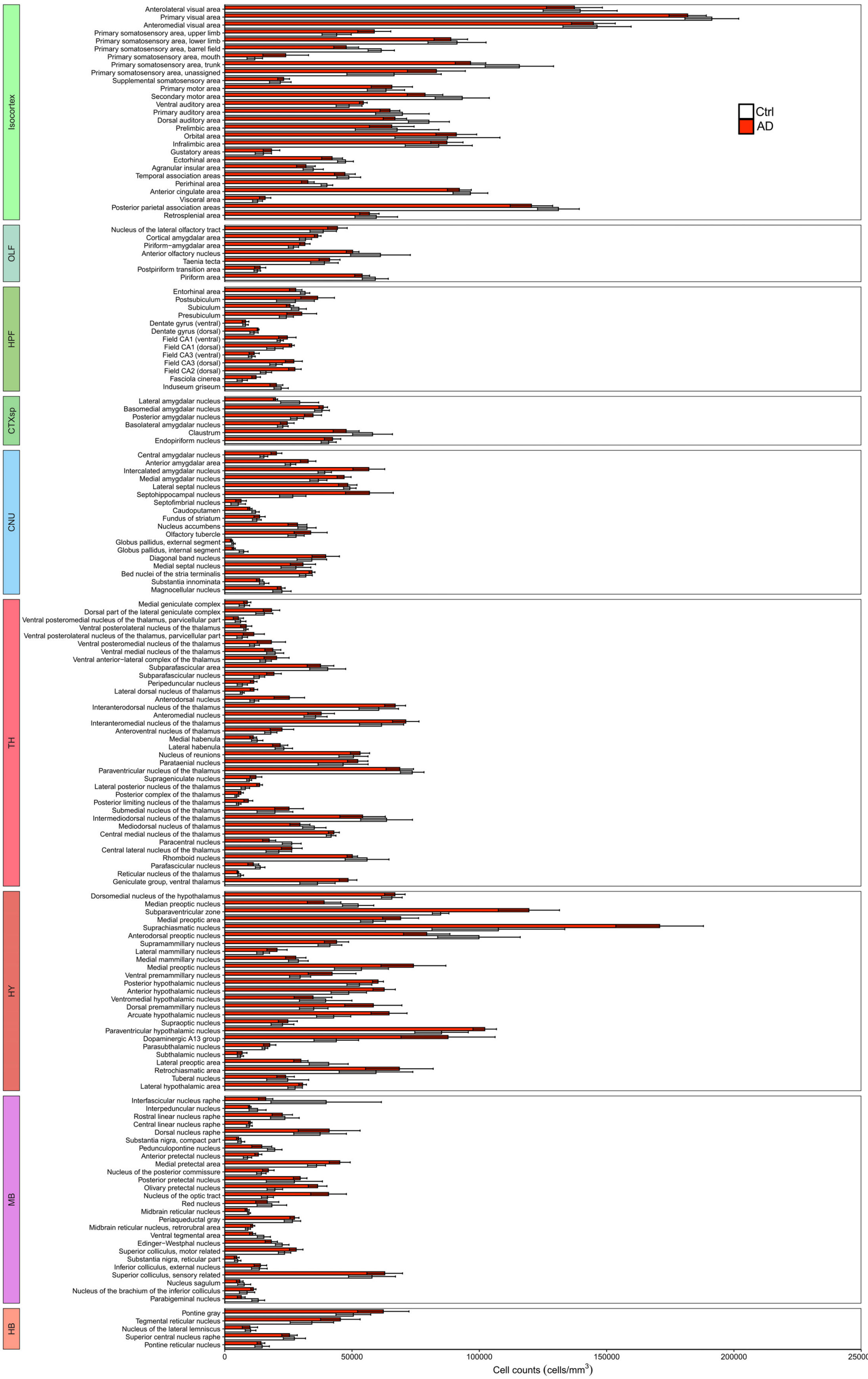

Supplementary Fig. 5 | Differential expression of c-Fos^{+} activity across all mapped regions. Volume normalized (cells / mm^{3}) counts of sunrise-active c-Fos^{+} cells between Ctrl (white bars) or AD

(green bars) mice across all regions mapped. Subregion activity expression patterns are organized by parent anatomical divisions. olfactory areas, OLF; hippocampal formation, HPF; cortical subplate, CTXsp; cerebral nuclei, CNU; thalamus, TH; hypothalamus, HY; midbrain, MB; hindbrain, HB. (n = 6-8 mice per group). Error bars represent ± SEM.

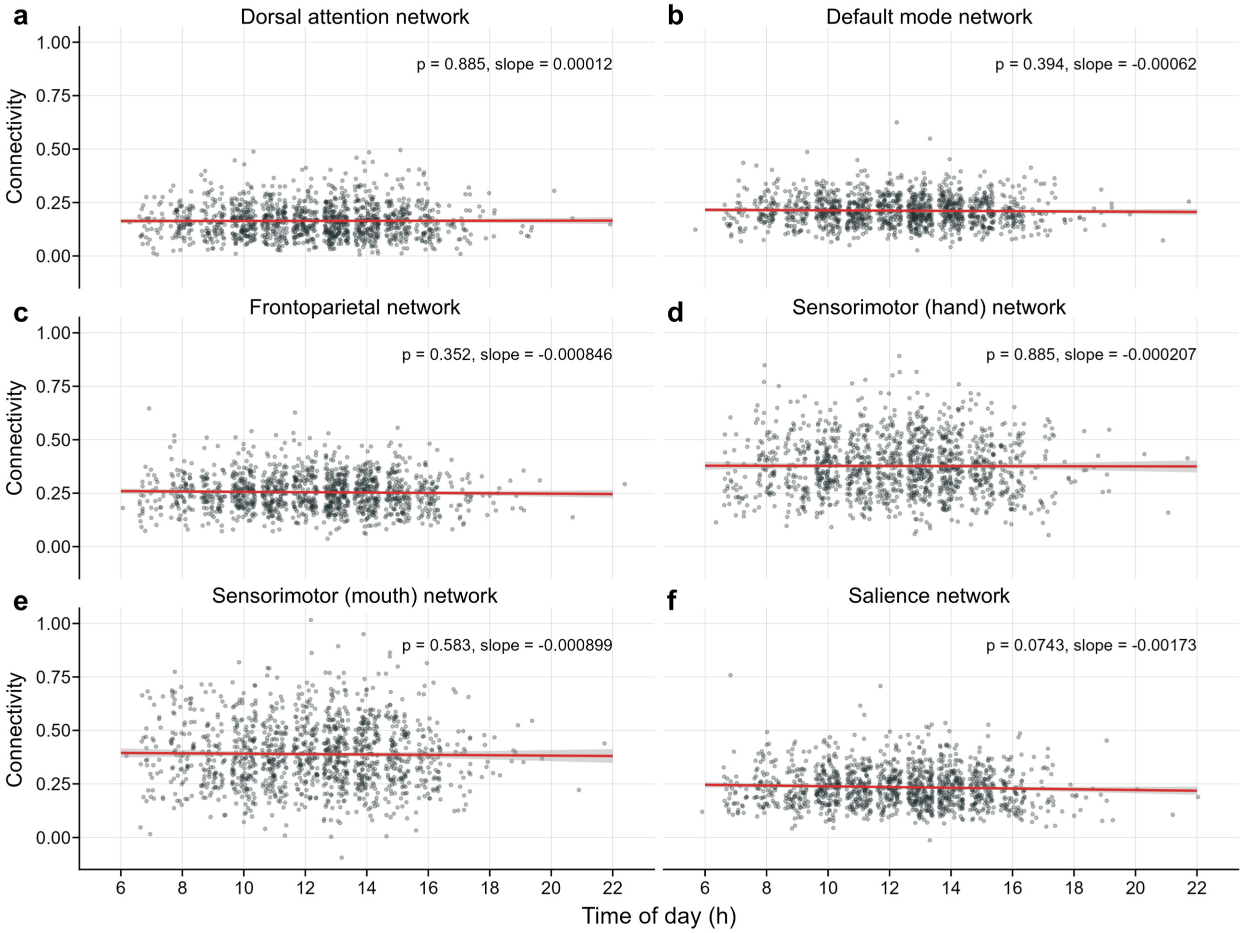

Supplementary Fig. 6. | Network connectivity over time of day does not change across most functional brain networks. (a-f) Resting-state functional connectivity does not change over time of day across all subjects (pooled across cognitive states) for the **(a)** dorsal attention network, **(b)** default mode network, **(c)** frontoparietal network**, (d)** sensorimotor (hand) network, **(e)** sensorimotor (mouth) network, and the **(f)** salience network. Individual values per scan are plotted as grey dots. The red lines represent the least-squares regression line per network.

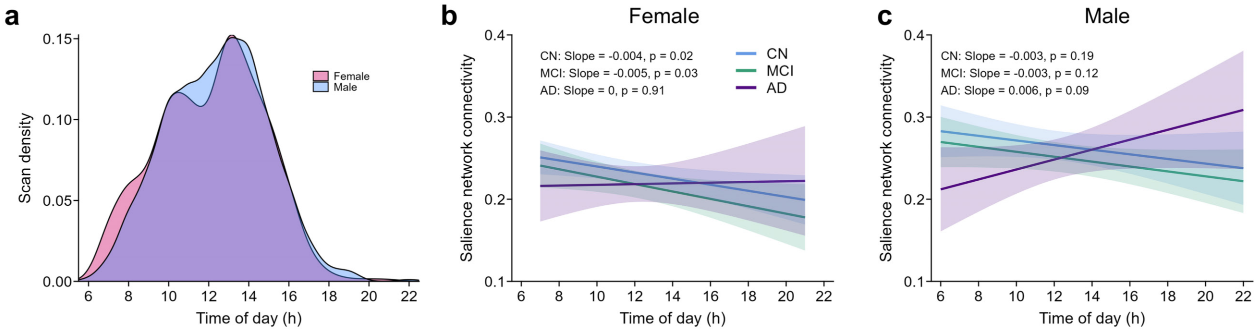

Supplementary Fig. 7 | Sex stratified analysis of salience network connectivity over time of day across cognitive conditions. (a) A density plot of showing the distribution of scan times in female subjects (pink) and male subject (blue). **(b-c)** Functional connectivity of the salience network in **(b)** female and **(c)** male CN, MCI, and AD subjects across time of day. Shaded portions denote the 95% confidence interval (CI). There is an interaction effect between cognitive status (AD diagnosis) with salience network connectivity over time of day in male (AD diagnosis x Time, p = 0.034), but not female subjects (AD diagnosis x Time, p = 0.300). Assessed using a mixed linear model controlling for several covariates (**b, c,** see **Methods**).
